# Genome-wide association study identifies human genetic variants associated with fatal outcome from Lassa fever

**DOI:** 10.1038/s41564-023-01589-3

**Published:** 2024-02-07

**Authors:** Dylan Kotliar, Siddharth Raju, Shervin Tabrizi, Ikponmwosa Odia, Augustine Goba, Mambu Momoh, John Demby Sandi, Parvathy Nair, Eric Phelan, Ridhi Tariyal, Philomena E. Eromon, Samar Mehta, Refugio Robles-Sikisaka, Katherine J. Siddle, Matt Stremlau, Simbirie Jalloh, Stephen K. Gire, Sarah Winnicki, Bridget Chak, Stephen F. Schaffner, Matthias Pauthner, Elinor K. Karlsson, Sarah R. Chapin, Sharon G. Kennedy, Luis M. Branco, Lansana Kanneh, Joseph J. Vitti, Nisha Broodie, Adrianne Gladden-Young, Omowunmi Omoniwa, Pan-Pan Jiang, Nathan Yozwiak, Shannon Heuklom, Lina M. Moses, George O. Akpede, Danny A. Asogun, Kathleen Rubins, Susan Kales, Anise N. Happi, Christopher O. Iruolagbe, Mercy Dic-Ijiewere, Kelly Iraoyah, Omoregie O. Osazuwa, Alexander K. Okonkwo, Stefan Kunz, Joseph B. McCormick, S. Humarr Khan, Anna N. Honko, Eric S. Lander, Michael B. A. Oldstone, Lisa Hensley, Onikepe A. Folarin, Sylvanus A. Okogbenin, Stephan Günther, Hanna M. Ollila, Ryan Tewhey, Peter O. Okokhere, John S. Schieffelin, Kristian G. Andersen, Steven K. Reilly, Donald S. Grant, Robert F. Garry, Kayla G. Barnes, Christian T. Happi, Pardis C. Sabeti

**Affiliations:** 1 Broad Institute of Massachusetts Institute of Technology (MIT) and Harvard, Cambridge, MA USA; 2 Department of Systems Biology, Harvard Medical School, Boston, MA USA; 3 Department of Internal Medicine, Brigham and Women’s Hospital, Boston, MA USA; 4 Department of Radiation Oncology, Massachusetts General Hospital, Boston, MA USA; 5 Koch Institute for Integrative Cancer Research, Massachusetts Institute of Technology, Cambridge, MA USA; 6 Institute of Lassa Fever, Research and Control, Irrua Specialist Teaching Hospital, Irrua, Nigeria; 7 College of Medicine and Allied Health Sciences, University of Sierra Leone, Freetown, Sierra Leone; 8 Eastern Polytechnic College, Kenema, Sierra Leone; 9 Howard Hughes Medical Institute, Chevy Chase, MD USA; 10 Prospr at Work Inc., Berlin, Germany; 11 NextGen Jane, Inc., Oakland, CA USA; 12 African Centre of Excellence for Genomics of Infectious Diseases (ACEGID), Redeemer’s University, Ede, Nigeria; 13 Department of Critical Care Medicine, University of Maryland Medical Center, Baltimore, MA USA; 14 Department of Immunology and Microbiology, The Scripps Research Institute, La Jolla, CA USA; 15 Equator Labs Incorporated, Washington, DC USA; 16 Biological Sciences Division, University of Chicago, Chicago, IL USA; 17 Department of Organismic and Evolutionary Biology, Harvard University, Cambridge, MA USA; 18 Department of Immunology and Infectious Diseases, Harvard T.H. Chan School of Public Health, Harvard University, Boston, MA USA; 19 Vir Biotechnology, San Francisco, CA USA; 20 Genomics and Computational Biology, UMass Chan Medical School, Worcester, MA USA; 21 Program in Molecular Medicine, UMass Chan Medical School, Worcester, MA USA; 22 Department of Medicine, Massachusetts General Hospital, Boston, MA USA; 23 Zalgen Labs, Frederick, MD USA; 24 Viral Hemorrhagic Fever Program, Kenema Government Hospital, Ministry of Health and Sanitation, Kenema, Sierra Leone; 25 New York-Presbyterian Hospital-Columbia and Cornell, New York, NY USA; 26 Molecular Microbiology, Graduate School of Biomedical Sciences, Tufts University, Boston, MA USA; 27 Malaria Consortium, Abuja, Nigeria; 28 Google Medical Brain, Mountain View, CA USA; 29 Gene and Cell Therapy Institute, Mass General Brigham, Cambridge, MA USA; 30 San Francisco Community Health Center, San Francisco, CA USA; 31 Tulane University School of Public Health and Tropical Medicine, New Orleans, LA USA; 32 Department of Medicine, Ambrose Alli University, Ekpoma, Nigeria; 33 Department of Community Medicine, Ambrose Alli University, Ekpoma, Nigeria; 34 National Aeronautics and Space Administration, Houston, TX USA; 35 The Jackson Laboratory, Bar Harbor, ME USA; 36 Department of Medicine, Irrua Specialist Teaching Hospital, Irrua, Nigeria; 37 Institute of Microbiology, University Hospital Center and University of Lausanne, Lausanne, Switzerland; 38 UTHealth Houston School of Public Health, Brownsville Campus, Brownsville, TX USA; 39 Boston University School of Medicine, Boston, MA USA; 40 Department of Biology, Massachusetts Institute of Technology (MIT), Cambridge, MA USA; 41 National Institutes of Health Integrated Research Facility, Frederick, MA USA; 42 Department of Biological Sciences, Redeemer’s University, Ede, Nigeria; 43 Bernhard Nocht Institute for Tropical Medicine, Hamburg, Germany; 44 Institute for Molecular Medicine Finland (FIMM), University of Helsinki, Helsinki, Finland; 45 Center for Genomic Medicine, Massachusetts General Hospital, Boston, MA USA; 46 Anesthesia, Critical Care, and Pain Medicine, Massachusetts General Hospital and Harvard Medical School, Boston, MA USA; 47 Section of Infectious Disease, Department of Pediatrics, Tulane University School of Medicine, New Orleans, LA USA; 48 Department of Genetics, Yale School of Medicine, New Haven, CT USA; 49 Tulane University School of Medicine, New Orleans, LA USA; 50 Malawi-Liverpool-Wellcome Trust Clinical Research Programme, Kamuzu University of Health Sciences, Blantyre, Malawi; 51 Department of Vector Biology and Tropical Disease Biology, Liverpool School of Tropical Medicine, Liverpool, UK; 52 Massachusetts Consortium on Pathogen Readiness, Boston, MA USA

**Keywords:** Genome-wide association studies, Virus-host interactions, Medical genomics

## Abstract

Infection with Lassa virus (LASV) can cause Lassa fever, a haemorrhagic illness with an estimated fatality rate of 29.7%, but causes no or mild symptoms in many individuals. Here, to investigate whether human genetic variation underlies the heterogeneity of LASV infection, we carried out genome-wide association studies (GWAS) as well as seroprevalence surveys, human leukocyte antigen typing and high-throughput variant functional characterization assays. We analysed Lassa fever susceptibility and fatal outcomes in 533 cases of Lassa fever and 1,986 population controls recruited over a 7 year period in Nigeria and Sierra Leone. We detected genome-wide significant variant associations with Lassa fever fatal outcomes near *GRM7* and *LIF* in the Nigerian cohort. We also show that a haplotype bearing signatures of positive selection and overlapping *LARGE1*, a required LASV entry factor, is associated with decreased risk of Lassa fever in the Nigerian cohort but not in the Sierra Leone cohort. Overall, we identified variants and genes that may impact the risk of severe Lassa fever, demonstrating how GWAS can provide insight into viral pathogenesis.

## Abstract (web summary)

GWAS in difficult-to-recruit populations identifies variants associated with Lassa fever outcome and susceptibility at loci proximal to LIF, GRM7 and LARGE1.

## Main

Lassa fever is an illness that can result from infection with Lassa virus (LASV). Initial Lassa fever symptoms (fever, vomiting, cough, sore throat) can quickly progress to respiratory distress, mucosal bleeding, shock and multiorgan failure^1^. Overall case fatality rates (CFRs) are as high as 29.7% in laboratory-confirmed patients^2^ and more than 50% in fetuses^3,4^. This lethality, coupled with the aerosol-based route of exposure and lack of approved therapeutics or vaccines, means that LASV is a World Health Organization risk group 4 pathogen, biosafety level 4 (BSL-4) agent and substantial threat to public health.

LASV is ubiquitous in many regions of West Africa. The main host and reservoir of LASV is *Mastomys natalensis*, a rodent that lives near houses in rural villages. Capture surveys have detected LASV in 3.2–52% of rodents^2,5^. LASV is transmitted to humans through aerosolization of viral particles from rodent excrement. Consistent with the rodent reservoir’s prevalence and virus’ transmissibility, antibody surveys indicate that between 8% and 52% of residents in some regions have been exposed to LASV^6,7^, leading to an estimated 100,000–300,000 infections of LASV annually^8^. Person-to-person transmission has been reported but usually only in nosocomial settings^9^.

Despite the prevalence of LASV, only hundreds to thousands of cases of Lassa fever are diagnosed each year^10^, suggesting that most infections are undocumented and mild. Why severe disease and death only occurs in a subset of LASV infections is not clear. Although old age^11^ and pregnancy^2,3^ are associated with poor Lassa fever outcomes, they do not explain all the variability in infection outcome. Variability among LASV lineages^12^ has not been linked to severity of symptoms.

**Fig. 1:**
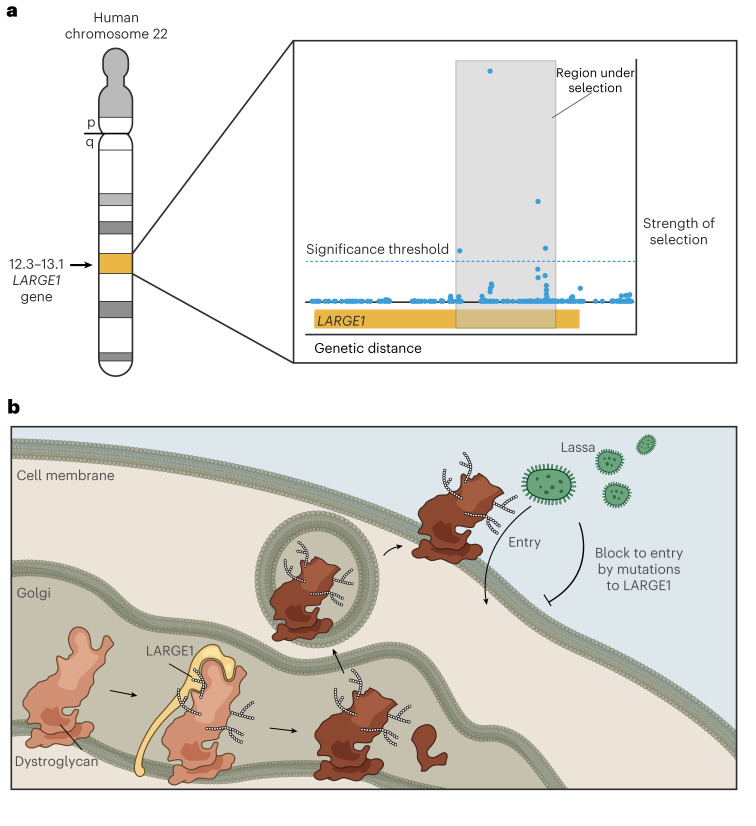
Overview of hypothesized mechanism of positive selection for resistance to Lassa fever mediated by *LARGE1*. **a**, Statistical evidence for positive selection at the *LARGE1* locus, adapted from Andersen et al.^17^. The *y* axis shows the composite likelihood score which integrates evidence of positive selection based on population differentiation (fixation index), long haplotype (integrated haplotype score, delta integrated haplotype score, cross-population extended haplotype homozygosity) and derived allele frequency. On the figure, p refers to the short arm of the chromosome, while q refers to the long arm. See Andersen et al.^17^ for details. **b**, Hypothesized mechanism by which decreased activity of *LARGE1* increases resistance to LASV infection and Lassa fever.

Human genetic variation may contribute to variability in the outcome of LASV infection. Host genetics has been linked to symptoms caused by infection with severe acute respiratory syndrome coronavirus 2, human immunodeficiency virus (HIV), dengue and hepatitis A–C^13–15^. The link between host genetics and LASV infection is intriguing because LASV may have been an important selective force in endemic regions, driving variants that protect against Lassa fever to higher prevalence. We previously reported a signal of positive selection in a Yoruba population from Nigeria, who live in a LASV endemic region, at a locus overlapping the gene *LARGE1* (refs. ^16,17^) (Fig. 1a). *LARGE1* encodes a protein that glycosylates α-dystroglycan, the primary cellular receptor for LASV^18,19^. LASV infectivity in vitro depends on the level of *LARGE1* expression^19^. Therefore, a variant in the putative region under positive selection may have been driven to high allele frequencies by impacting expression levels of *LARGE1*, thereby reducing the risk of severe Lassa fever (Fig. 1b). Given Lassa fever’s lethality among diagnosed cases and the high seroprevalence to LASV, it is plausible that host variants providing resistance might have an impact on reproductive fitness. In addition, phylogenetic dating indicates that LASV has been present for over 1,000 years in Nigeria^12^, making it feasible that the virus might have exerted evolutionary pressure on humans. However, no previous studies have systematically assessed the impact of host variation in LASV infection.

Despite the clinical importance of Lassa fever, there are practical obstacles to studying it in human patients. First, LASV is a BSL-4 pathogen endemic in countries that have only recently obtained infrastructure for safe virus handling. Second, medical infrastructure is lacking in the villages where Lassa fever is most common, so most symptomatic Lassa fever cases are undocumented. Finally, genetic diversity of LASV isolates means that diagnostics based on nucleic acid amplification or immunoassays can have low sensitivity. As there are no US Food and Drug Administration-approved LASV diagnostics^20^, proven diagnoses require viral culture, which is generally not feasible. We anticipated that it would be challenging to obtain a sizable enough cohort to carry out a Lassa fever genome-wide association study (GWAS) but hypothesized that increased power would arise if natural selection for resistance to Lassa fever was present. This is because natural selection would increase the prevalence of advantageous alleles, over time generating common resistance alleles. Such highly protective variants might be detectable in genetic association studies of modest sample size. For instance, the sickle cell allele in haemoglobin is one of the most robust signals of genetic resistance to infectious disease and can be detected in small samples^21,22^. We hypothesized that if this was the case, a Lassa fever GWAS could elucidate the biological basis of Lassa fever resistance.

Beginning in 2008, we established public health and research capabilities for Lassa fever in two countries in West Africa. To obtain an adequate cohort size, we recruited and genotyped patients with Lassa fever and geographically matched individuals who do not have LASV symptoms (population controls) during a 7 year period from LASV endemic regions of Nigeria and Sierra Leone using an array of diagnostic tests to capture the broadest possible set of cases while minimizing false positives. We tested for genome-wide association with Lassa fever susceptibility and fatal outcomes, with sub-analyses specifically considering variation at *LARGE1* and the human leukocyte antigen (HLA) loci.

## GWAS recruitment and clinical characterization

**Extended Data Fig. 1:**
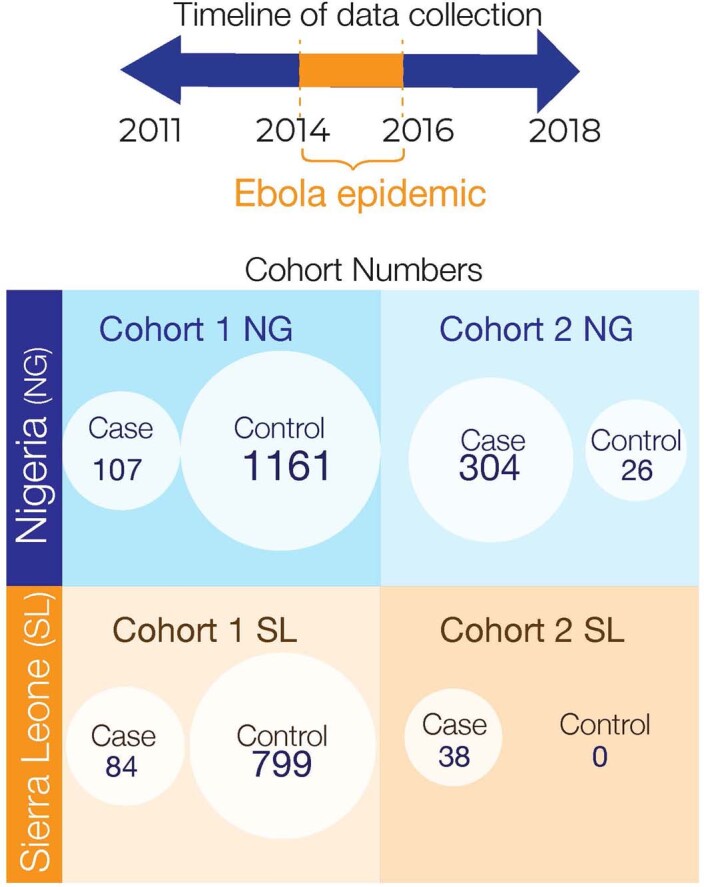
Timeline of cohort recruitment in each country. Breakdown of enrolled patients by country, cohort, and disease status.

We recruited and genotyped 411 people with LASV and 1,187 controls from Nigeria and 122 people with LASV and 799 controls from Sierra Leone (Extended Data Table 1 and Extended Data Fig. 1).

We used the standard-of-care assays for case definition at each recruitment site and also used next-generation sequencing to detect additional people with LASV missed by traditional diagnostics (Supplementary Note and Extended Data Table 2).

All sequenced LASV genomes from Nigeria were clade II or III, and those from Sierra Leone were clade IV, matching the expected distributions^23^. Furthermore, all but one of the Nigeria genomes matched the expected phylogeographic distribution of clade III samples deriving from northern Nigeria and clade II samples deriving from southern Nigeria^24^.

**Fig. 2:**
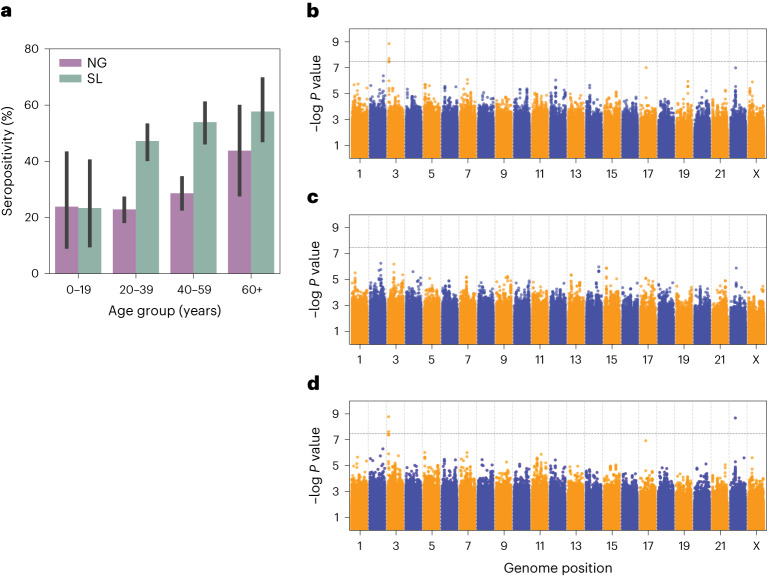
GWAS of Lassa fever clinical outcome. **a**, Immunoglobulin G seropositivity rate in Nigerian (NG) and Sierra Leonean (SL) controls stratified by age. Error bars represent 95% bootstrap confidence intervals. NG: *N* of 24 in 0–19 years, 424 in 20–39 years, 269 in 40–59 years and 34 in 60+ years. SL: *N* of 33 in 0–19 years, 282 in 20–39 years, 191 in 40–59 years and 83 in 60+ years. **b**–**d**, Manhattan plots showing the −log *P* value for each genomic variant for the Lassa fever outcome association for Nigeria (**b**), Sierra Leone (**c**) and meta-analysis (**d**). *P* values for **b** and **c** are based on SAIGE, while *P* values for **d** are derived from meta-analysis (METAL) of *P* values shown in **b** and **c**.

As we recruited population controls from Lassa fever endemic villages, we suspected that many controls were exposed to LASV in their lifetimes but never developed clinically relevant Lassa fever, thus increasing their likelihood of harbouring protective genetic variation. We used enzyme-linked immunosorbent assays (ELISAs) to measure immunoglobulin G antibodies against LASV for 751 and 589 of the controls from Nigeria and Sierra Leone, respectively (Supplementary Note). We found that 25.9% and 49.6% of the Nigeria and Sierra Leone controls were seropositive, respectively (compared to 0/117 of United States-based controls^25^), consistent with the upper end of previous seroprevalence surveys in these countries^6^. Furthermore, we found that seropositivity was associated with older age (rank-sum test *P* = 0.0022 for Nigeria and 0.00053 for Sierra Leone) and increased gradually with age (Fig. 2a), suggesting continuous lifetime exposure to LASV.

**Extended Data Fig. 2:**
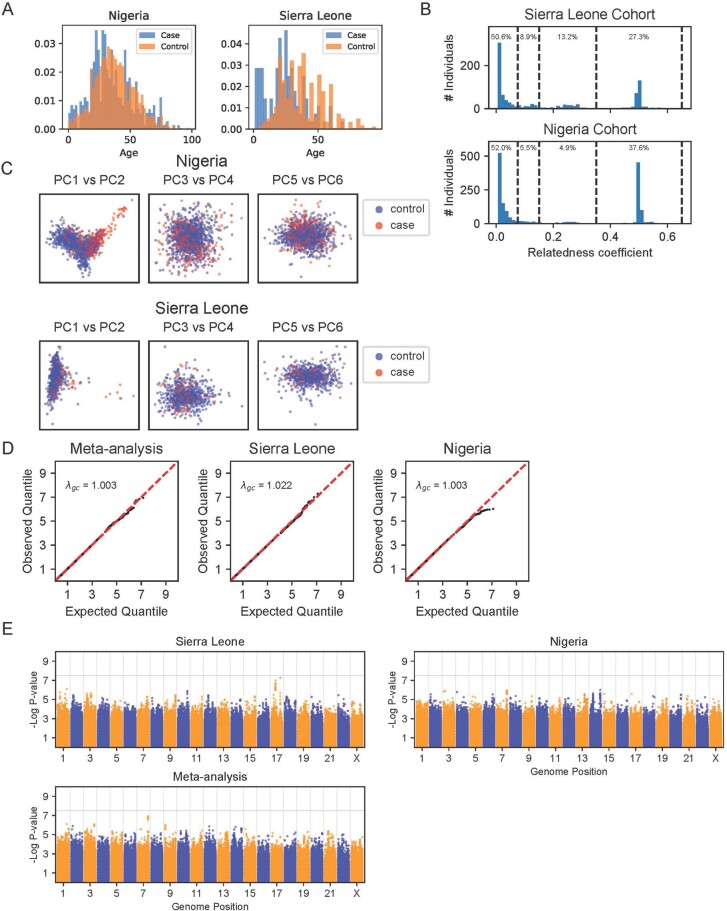
Quality control analyses for the susceptibility GWAS. (**A**) Histogram of ages in the Nigeria and Sierra Leone cohorts, separated by case/control status. (**B**) Histogram of the maximum relatedness coefficient between each individual and all other individuals in the Nigerian (NG) and Sierra Leonean (SL) cohorts. (**C**) Principal component analysis (PCA) of the NG and SL cohorts, colored by case-control status. PCs were computed on unrelated individuals and then all individuals were projected onto those components (Methods). (**D**) Quantile-quantile plots of -log10 P-values from the susceptibility GWAS against expected quantiles. (**E**) Manhattan plots showing the -log10 P-value for each genomic variant for the LF susceptibility associations. P-values in D and E are based on saddlepoint-approximated score tests (SAIGE), while meta-analysis P-values are derived from meta-analysis (METAL) of P-values generated from each cohort.

We tested whether demographic variables were associated with Lassa fever susceptibility and fatal outcomes. Previous studies reported higher proportions of women and girls with Lassa fever^26–32^, suggesting increased susceptibility to LASV or exposure to LASV among women^32,33^. Consistent with this, we found that women and girls are significantly overrepresented within our Nigeria cases (242/411 or 58.9%, binomial test *P* = 0.0003). However, we did not find significant sex differences in the Sierra Leone cases (50/122 or 41.0%, *P* = 0.057). We found that people with LASV were younger than controls in both Nigeria and Sierra Leone (rank-sum test *P* = 0.0010 and 2.15 × 10^−17^, respectively) (Extended Data Fig. 2a). CFR was estimated to be 35.3% and 64.8% in our Nigeria and Sierra Leone cases, respectively, consistent with previous estimates in these countries^2^ (Extended Data Table 1).

We tested the association between symptoms and age (Extended Data Table 3) and found that younger patients in both Nigeria and Sierra Leone were more likely to present with vomiting (*P* = 0.016 and 0.012, respectively) and cough (*P* = 0.08 and 0.001, respectively) than older patients. We also observed a trend toward higher probability of fatal outcome in older people with LASV, but this was not significant (*P* = 0.11 and 0.17, respectively, in Nigeria and Sierra Leone).

## GWAS of Lassa fever susceptibility and clinical outcome

Owing to the prolonged, interrupted recruitment over 7 years and changes in genotyping platforms over the time frame of recruitment, samples were genotyped on three different arrays: H3Africa, Omni 2.5 M and Omni 5 M (Extended Data Table 2). We corrected for array-derived batch effects before joint imputation across all arrays (Supplementary Note). This yielded a pre-imputation set of 1,453,101 genotyped variants and a final imputed set of 12,783,971 variants in Nigeria and 12,522,562 variants in Sierra Leone.

We used generalized linear mixed models as implemented in saddlepoint-approximated score tests (SAIGE)^34^ to account for relatedness and population stratification in our dataset (Methods). Mixed models analysis is important for this study because the dataset contained many first-degree relatives. Six hundred and sixteen (38%) and 251 (27%) individuals in the Nigerian and Sierra Leone cohorts had a first-degree relative, respectively (Extended Data Fig. 2b). In addition, principal component analysis showed evidence of stratification even after removing closely related individuals in our cohort (Extended Data Fig. 2c); we therefore included principal components (PCs) as fixed effects, which has been shown to control for confounding due to population stratification^35^. We used a genome-wide significance threshold of 3.24 × 10^−8^ (previously reported to control for false positives in African populations^36^). Quantile–quantile plots did not show any evidence of test-statistic inflation, indicating that our statistical controls accounted for dominant confounding variables (Extended Data Fig. 2d).

A GWAS of susceptibility to Lassa fever infection for all individuals in our study did not identify any variants that reached genome-wide significance in either cohort. However, two variants on chromosome 17 showed a trend toward significance in the Sierra Leone cohort (Table 1 and Extended Data Fig. 2e). rs73397758 (*P* = 5.5 × 10^−8^, odds ratio (OR) = 9.16) is ~350 KB (kilobase pairs) downstream of the gene *CASC17*, a long non-coding RNA named for a genetic association with prostate cancer^37^, and 570 KB upstream of *KCNJ2*, a potassium inwardly rectifying channel^38^. rs143130878 (*P* = 1.1 × 10^−7^, OR = 6.87) resides 62,472 base pairs downstream of the gene *CCT6B*^39^, which is a member of the molecular chaperone (TRiC) family that has been shown to regulate the replication of arenaviruses, including LASV^40^. Neither variant was significantly associated with susceptibility in the Nigeria cohort (*P* = 0.58 and *P* = 0.64, respectively).

**Table 1:** Description of lead variants for the susceptibility GWAS analysis

Lead SNP												
Lead SNP	Chromosome	Position (hg19)	Nearest gene	Nigeria OR	Nigeria 95% CI	Nigeria *P* value	Nigeria MAF (%)	Sierra Leone OR	Sierra Leone 95% CI	Sierra Leone *P* value	Sierra Leone MAF (%)	Meta-analysis *P* value
rs114992845	7	146356694	*CNTNAP2*	9.19	[3.5, 23.9]	2.7 × 10^−6^	1.21	4.77	[1.3, 17.8]	0.010	1.86	1.2 × 10^−7^
rs143130878	17	33192408	*CCT6B*	1.20	[0.6, 2.6]	0.64	3.38	6.87	[3.3, 14.2]	1.1 × 10^−7^	2.74	3.3 × 10^−4^
rs73397758	17	68745251	*CASC17*	0.84	[0.5, 1.5]	0.58	6.28	9.16	[4.0, 20.8]	5.5 × 10^−8^	2.42	4.8 × 10^−3^

Includes the most significant variant in the meta-analysis of both cohorts and the two most significant variants in the Sierra Leone analysis. Country-specific *P* values are based on SAIGE, while meta-analysis *P* values are derived from meta-analysis (METAL) of *P* values generated from each cohort. 95% CI, 95% confidence interval for the OR; MAF, minor allele frequency.

The most significant variant in a meta-analysis of the two GWAS cohorts was rs114992845 in an intron of *CNTNAP2* (meta-analysis *P* = 1.2 × 10^−7^; Nigeria OR = 9.19, Sierra Leone OR = 4.77) (Table 1). *CNTNAP2* is a member of the neurexin family, many members of which encode proteins that bind to α-dystroglycan, the cellular receptor for LASV^41^. Furthermore, loss-of-function mutations in the gene *CNTNAP2* have been associated with recurrent infections^42^, although the underlying mechanism remains unknown. All three variants that were trending toward significance in the susceptibility GWAS are of low frequency (Table 1) and will require larger sample sizes for validation.

**Extended Data Fig. 3:**
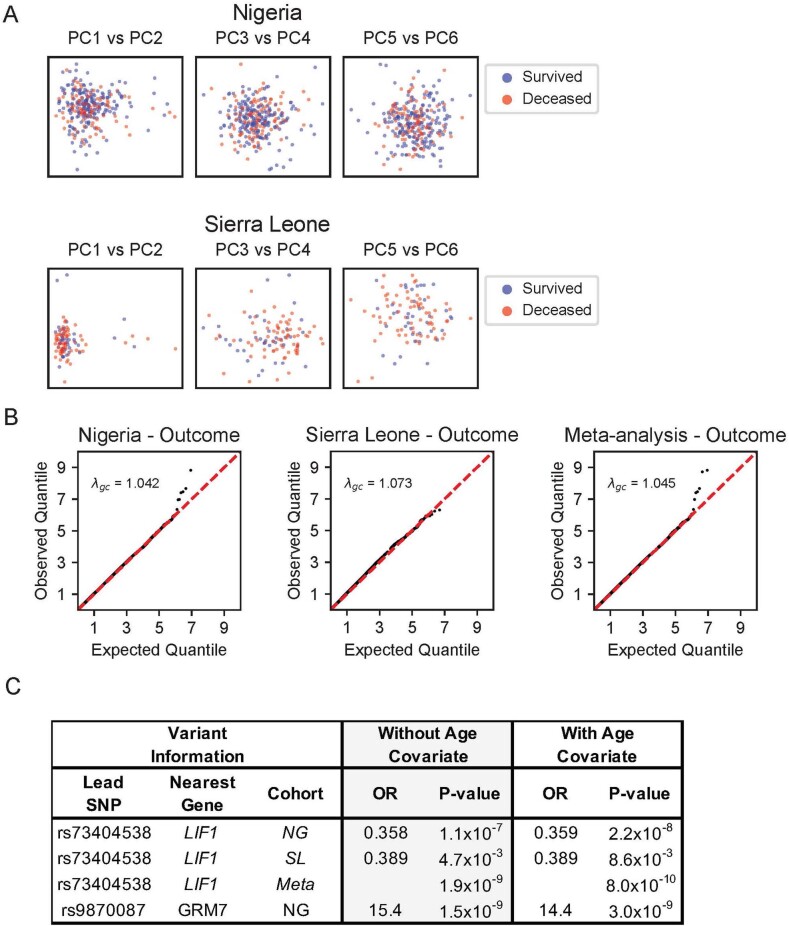
Quality control analyses for the GWAS of LF clinical outcome. (**A**) Principal component analysis (PCA) of the NG and SL cohorts, colored by clinical outcome. PCs were computed on unrelated individuals, and then all individuals were projected onto those components. (**B**) Quantile-quantile plots of -log10 P-values from the outcome GWAS against expected quantiles. (**C**) Comparison of the outcome GWAS lead variants with and without inclusion of age as a covariate. P-values in B and C are based on saddlepoint-approximated score tests (SAIGE), while meta-analysis P-values are derived from meta-analysis (METAL) of P-values generated from each cohort. Odds ratios are computed from Firth logistic regression.

A GWAS of fatal outcomes in Lassa fever cases using the same strategy described above did identify genome-wide significant associations (Extended Data Fig. 3a). We did not observe evidence of population stratification or test statistic inflation (Supplementary Fig. 3a,b). We identified a significant association with rs9870087 in the Nigeria cohort, falling within an intron of the gene *GRM7* (*P* = 1.54 × 10^−9^, OR = 15.4) (Table 2 and Fig. 2b). The protein encoded by *GRM7* is a glutamate metabotropic receptor active throughout the central nervous system^43^. While no direct role of this receptor is known in viral infection, *GRM2*, another member of this family, has been previously linked to severe acute respiratory syndrome coronavirus 2^44^ and rabies^45^ viral entry. A recent *GRM7* knock-out mouse implicated this gene in neuroimmune signalling in anaphylaxis^46^. Furthermore, *GRM7* has an important role in maintenance of hearing by inner-ear hair cells^47^, and hearing loss is a symptom of Lassa fever^48^. We did not identify any genome-wide significant associations in the Sierra Leone cohort (Fig. 2c).

**Table 2:** Description of lead variants for the fatal outcome GWAS analysis

Lead SNP												
Lead SNP	Chromosome	Position (hg19)	Nearest gene	Nigeria OR	Nigeria 95% CI	Nigeria *P* value	Nigeria MAF (%)	Sierra Leone OR	Sierra Leone 95% CI	Sierra Leone *P* value	Sierra Leone MAF (%)	Meta-analysis *P* value
rs73404538	22	30619983	*LIF*	0.358	[0.2, 0.5]	1.1 × 10^−7^	47.8	0.389	[0.19, 0.79]	4.7 × 10^−3^	35.8	1.9 × 10^−9^
rs9870087	3	7330265	*GRM7*	15.4	[6.2, 37.9]	1.5 × 10^−9^	4.73	0.642^a^	[0.1, 2.8]^a^	0.55^a^	5.02^a^	1.1 × 10^−6a^

Includes the most significant variant per genomic locus containing at least one genome-wide significant association (including in meta-analysis). *P* values are based on SAIGE, while meta-analysis *P* values are derived from meta-analysis (METAL) of *P* values generated from each cohort.

^a^rs9870087 was excluded from the Sierra Leone GWAS due to low minor allele count but is included here for completeness.

We also carried out a meta-analysis of fatal outcomes in the Nigeria and Sierra Leone cohorts which identified a genome-wide significant association with rs73404538 (meta-analysis *P* = 1.9 × 10^−9^; Nigeria OR = 0.358, Sierra Leone OR = 0.389) (Fig. 2d and Extended Data Table 4). This variant falls 16,453 base pairs downstream of the 3′ untranslated region of *LIF*, which encodes an interleukin 6 class cytokine^49^ that has been associated with several viral infections. We further note that rs73404538 is nominally significant in the Sierra Leone susceptibility GWAS (*P* = 0.039, OR = 0.71) and in a meta-analysis of the Nigeria and Sierra Leone susceptibility GWASs (*P* = 0.021) with a concordant direction of effect (Extended Data Table 4). This suggests that in addition to increasing the lethality of Lassa fever, rs73404538 may also increase the probability of contracting clinically detected Lassa fever.

We did not include age as a covariate in our primary analysis due to missing data for many participants (2.4% of Nigeria cases and 25.5% of Sierra Leone controls), but we did so in a secondary analysis. While the *P* values for the susceptibility lead variants decrease by up to 1 order of magnitude, consistent with a loss of power from the decreased sample size, the rs73404538 variant downstream of *LIF* actually becomes genome-wide significant in the Nigeria cohort (*P* = 2.2 × 10^−8^, OR = 0.36) and more significant in the meta-analysis (*P* = 8.0 × 10^−10^) providing further support for this association (Extended Data Fig. 3c).

**Extended Data Fig. 4:**
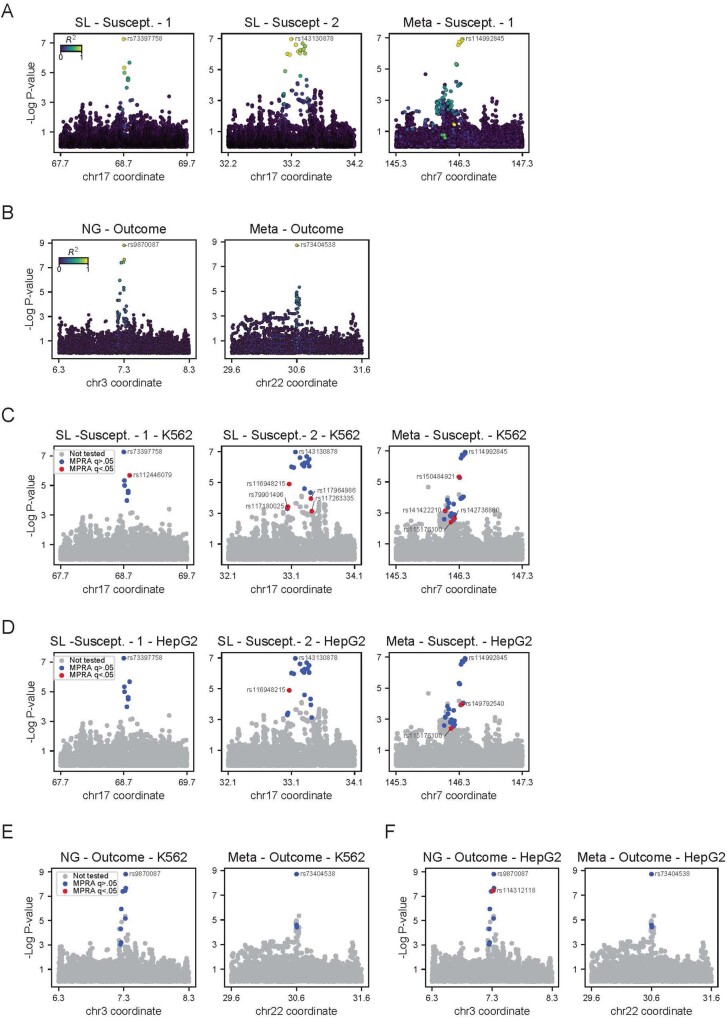
MPRA analyses of the susceptibility and outcome GWAS peaks. (**A**) Scatter plot of lead susceptibility GWAS loci described in the main text showing chromosomal position against -log10 association P-value. Variants are colored by the linkage disequilibrium (LD) coefficient of determination R^2^ between each variant and the most significant ‘lead’ variant in the locus. (**B**) Same as A but for the lead variants in the fatal outcome GWAS. (**C**–**F**) Same as A and B but colored by whether the variant showed statistically significant skew (q-value < 0.05) in the massively parallel reporter assay in the K562 cell line (**C** and **E**) or HepG2 cell line (**D** and **F**). P-values are based on saddlepoint-approximated score tests (SAIGE), while meta-analysis P-values are derived from meta-analysis (METAL) of P-values generated from each cohort.

As each of the candidate GWAS loci described above contains multiple linked non-coding genetic variants (Extended Data Fig. 4a,b), we used a massively parallel reporter assay (MPRA) to identify which variants are most likely to be functional. MPRA^50^ identifies potential regulatory variants by testing the reference and alternate alleles of thousands of variants in parallel for their ability to impact expression of a plasmid-based reporter (Supplementary Note). We carried out MPRA in K562 and HepG2 cells for loci containing the most significant variants in the susceptibility and fatal outcome GWASs (Supplementary Tables 3–5).

We identified potential regulatory variants in many of our top GWAS loci. For the *CASC17* locus, we find that the only tested variant to show regulatory activity is rs112446079 in K562 cells (log_2_ skew = −0.64, *q* = 0.031), the second most strongly associated variant in the region (Extended Data Fig. 4c, left). Similarly, for the *CNTNAP2* locus, the seventh most strongly associated variant in the region, rs150484921, showed regulatory activity by MPRA (log_2_ skew = −0.65, *q* = 0.011), but the lead variant did not (Extended Data Fig. 4c, right). Several variants were associated with the second Sierra Leone peak near *CCT6B*, the most significant of which in the GWAS was rs116948215 (log_2_ skew = −0.98, *q* = 1.94 × 10^−6^). This latter single-nucleotide polymorphism (SNP) is active in the MPRA in HepG2 cells as well as K562s suggesting a broader regulatory effect across cell types (Extended Data Fig. 4c, middle). For the outcome analysis, we identified one potential regulatory variant at the *GRM7* locus, rs114312118, which is active specifically in HepG2s (log_2_ skew = 0.87, *q* = 0.0077) (Extended Data Fig. 4f).

## Analysis of a positive selection signal overlapping *LARGE1*

Next, we tested whether variation around the gene *LARGE1*, a required LASV entry factor, is associated with resistance to Lassa fever. Previous studies identified a long-range haplotype at this locus, that is, multiple genetic variants located up to 500 KB apart that remain in tight LD. The presence of such an extended haplotype suggests that one or more variants in the locus provides a fitness advantage, causing it to spread to high allele frequency in the population faster than genetic recombination would break down the haplotype^16,17^.

**Fig. 3:**
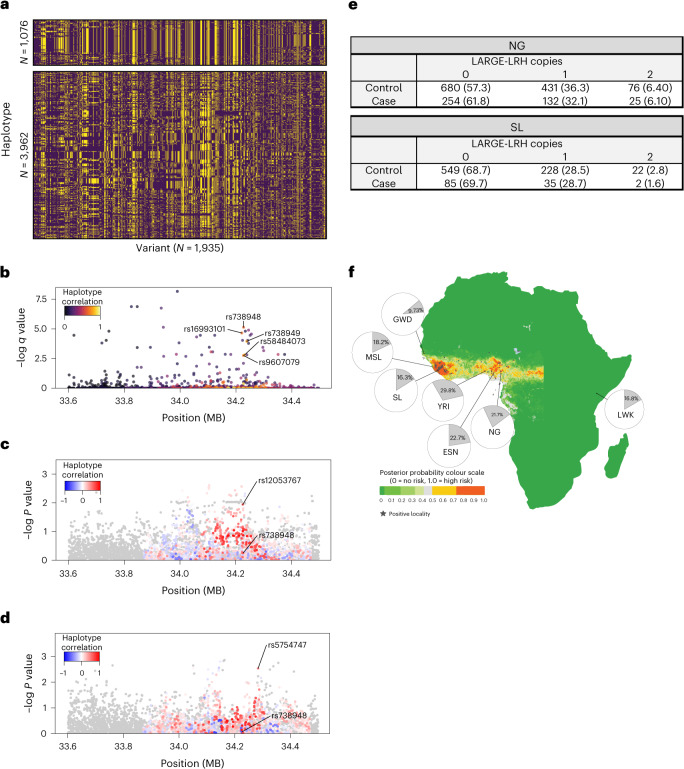
Association of the LARGE-LRH haplotype with susceptibility to Lassa fever. **a**, *K*-means clustering of haplotypes in the *LARGE1* region. Rows are phased haplotypes; columns are individual variants with reference alleles shown in purple, alternate alleles shown in yellow and *K*-means clusters separated. **b**, Scatter plot of *q* values for allelic skew in the MPRA, coloured by the absolute value of the Pearson correlation with the haplotype. **c**,**d**, Scatter plot of GWAS association *P* values over the *LARGE1* region for Nigeria (**c**) and Sierra Leone (**d**) coloured by Pearson correlation of the protective allele in the GWAS with the LARGE-LRH. *P* values in **c** and **d** are based on SAIGE. **e**, Contingency table of LARGE-LRH genotype counts in cases and controls for Nigeria (NG, top) and Sierra Leone (SL, bottom). **f**, Ecologically estimated Lassa fever prevalence from Fichet-Calvet et al.^70^ with pie charts indicating the frequency of the *LARGE1* haplotype in 1000 Genomes populations (YRI, Yoruba; ESN, Esan; MSL, Mende; LWK, Luhya; GWD, Gambian Mandinka)^51^ or our GWAS cohorts (NG, SL). Stars indicate towns, villages or hospitals that encountered outbreaks as detailed in Fichet-Calvet et al.^70^.

Although no individual variants on chromosome 22 reached genome-wide significance in the GWAS, we examined the long-range haplotype overlapping the *LARGE1* locus as a single entity to further characterize its correlation with Lassa fever phenotypes. We used *K*-means clustering (with *K* = 2) of phased haplotypes and found a dominant haplotype with long-range LD (Fig. 3a and Methods). We label this haplotype ‘*LARGE1* long-range haplotype’ or LARGE-LRH, for short. LARGE-LRH was well tagged by the lead variants identified in previous positive selection scans, for example, rs5999077, rs1013337 and rs1573662, identified in ref. ^16^ (*D*′ values of 0.957, 0.773 and 0.735). LARGE-LRH was present at 23.9% and 16.9% allele frequency in the Nigeria and Sierra Leone cohorts, respectively.

As LARGE-LRH comprises 96 tightly linked variants with Pearson correlation above 0.6 using the *K*-means annotation, we applied MPRA to zoom into potentially causal variants underlying the signal of positive selection. We tested a library of 5,286 oligonucleotides (of 200 base pair length) centred on different alleles of 1,674 variants in the *LARGE1* region for regulatory function using MPRA (Supplementary Note) (Fig. 3b). Fifty-four of the 1,674 tested variants (3.23%) had significant skew (false discovery rate (FDR)-adjusted *P* < 0.05) between the reference and alternate allele. Of these, five (rs738948, rs16993101, rs738949, rs58484073 and rs9607079) had an FDR-adjusted *P* < 0.01 and were linked to the haplotype with a Pearson correlation >0.6. This analysis shows that these variants might regulate gene expression and are candidates for positive selection effects in human populations.

We next evaluated whether any variants in linkage with LARGE-LRH were associated with susceptibility to Lassa fever (Fig. 3c,d). The haplotype-linked variant with the strongest association with Lassa fever susceptibility in the Nigeria cohort was rs12053767 (*P* = 0.011, haplotype Pearson correlation of 0.57). However, this variant was not significantly skewed by MPRA (*q* = 0.998) and was not significantly associated with Lassa fever in the Sierra Leone cohort (*P* = 0.25). The haplotype-linked variant with the strongest association to Lassa fever susceptibility in the Sierra Leone cohort was rs5754747 (*P* = 0.0030, haplotype Pearson correlation of 0.46), but this variant was also not significant in the Nigeria cohort (*P* = 0.988) or significantly skewed by MPRA (*q* = 0.26).

**Extended Data Fig. 5:**
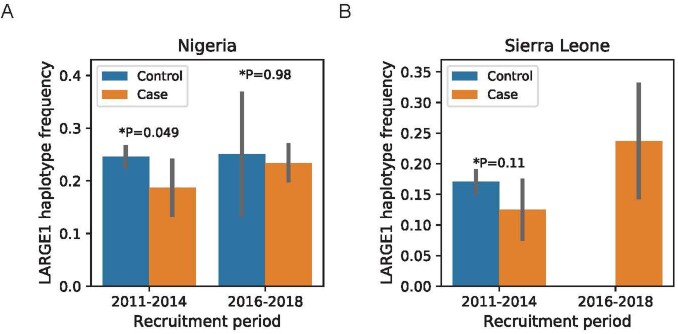
LARGE1 haplotype association by recruitment period. (**A**, **B**) Frequencies of the long-range LARGE1 haplotype by the period of recruitment as well as by case-control status for Nigeria (**A**) and Sierra Leone (**B**). P-values are from mixed logistic models association testing within the indicated recruitment period. Error bars represent 95% bootstrap confidence intervals for allele frequency. N for each cohort within each country is defined in Table S2.

We reasoned that LARGE-LRH, taken together as a single allele, could yield a stronger signal than individual SNPs if the causal variant is not genotyped or if the causal mechanism involves an interaction among multiple variants on the haplotype. We tested whether LARGE-LRH is associated with Lassa fever using the same model that we used in the primary GWAS and found that LARGE-LRH was significantly associated with Lassa fever susceptibility in Nigeria (*P* = 0.0492) but not in Sierra Leone (*P* = 0.412). The overall allele frequency of LARGE-LRH was slightly higher in controls than in people with LASV (Nigeria, 24.6% allele frequency in controls versus 22.1% in people with LASV; Sierra Leone, 17.0% versus 16.0%), consistent with our hypothesized resistance model (Fig. 3e). We note that the association with LARGE-LRH is mainly driven by individuals recruited in the first cohort (Nigeria 2011–2014 recruitment *P* = 0.049, Nigeria 2016–2018 recruitment *P* = 0.98) and that there is a trend toward association in the Sierra Leone cohort during that time period (Sierra Leone 2011–2014 recruitment *P* = 0.11). As there were no controls recruited in Sierra Leone in the second cohort, we do not have a 2016–2018 comparison for it. We were surprised that people with LASV recruited in 2016–2018 did not have a lower frequency of LARGE-LRH (Extended Data Fig. 5), so further study is necessary to harmonize these conflicting observations.

To further test the link between the selection signal at *LARGE1* and Lassa fever, we used 1000 Genomes Project (1KGP) data to test whether LARGE-LRH was present at higher frequency in populations living in LASV endemic regions. We quantified the haplotype frequency of individuals from 26 populations sequenced by the 1KGP^51^, including several African populations in LASV endemic regions (Esan, Yoruba and Mende) (Fig. 3f). We identified tag SNPs linked to the LARGE-LRH with Pearson correlation >0.92. We then analysed phased 1KGP sequence data and called the LARGE-LRH if three or more of the haplotype-linked alleles were present (Methods). The 1KGP cohort contained 27 individuals homozygous for the LARGE-LRH, 198 heterozygous individuals and 2,279 carrying 0 copies. LARGE-LRH was absent from all European and Asian ancestry populations tested and was present at the highest frequency in populations in LASV endemic regions (Yoruba 30.5%, Esan 23.2% and Mende 20.0%) (Fig. 3f). It was also present in Luhya (16.7%) and Mandinka (10.2%), African populations, outside of the LASV endemic zone (Fig. 3f). Mandinka are geographically close to the Lassa fever endemic region, and the Luhya are historically tied to West Africa through the Bantu expansion, so the elevated allele frequencies could be explained by migration after the putative selective sweep or by a changing geographic distribution of LASV.

## Imputation and association analysis of HLA alleles

We tested for associations between Lassa fever and genetic variation in the HLA region. HLA genes encode polymorphic proteins that present antigens to T cells and have been associated with many infectious disease phenotypes^15^. While we did not identify genome-wide significant associations with SNPs in the HLA genes, HLA-specific imputation approaches are frequently required to identify HLA associations^52^.

We imputed four-digit HLA alleles, which are complete amino acid sequences, and additional sequencing-based HLA typing of eight classical HLA genes to serve as ‘ground truth’ HLA calls to evaluate imputation accuracy (Methods). Sequencing-based typing of the eight classical HLA genes in 297 individuals in our Sierra Leone cohort identified 41 novel HLA alleles that were not present in the International Immunogenetics database (Extended Data Table 5). Nine of the novel alleles were from HLA class I loci, while 32 were HLA class II, with *DQB1* and *DPA1* having the most novel alleles with 11 and 9, respectively. Notably, a novel allele at 5% allele frequency, DPA1*03:01@2, disrupts the start codon (ATG to ACG).

**Fig. 4:**
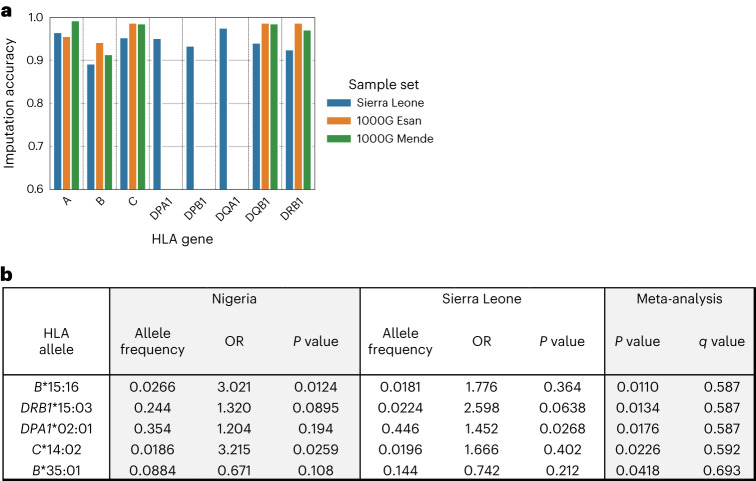
Association of HLA variation with Lassa fever susceptibility. **a**, Imputation accuracy of four-digit HLA calls compared to sequencing-based ground truth sets from our Sierra Leone cohort, as well as Esan and Mende individuals from 1000 Genomes. **b**, Table of HLA alleles with the strongest association with Lassa fever susceptibility, ordered by meta-analysis of the NG and SL cohorts. *P* values are based on SAIGE, while meta-analysis *P* values are derived from meta-analysis (METAL) of *P* values generated from each cohort. ORs are computed from Firth logistic regression.

We compared imputation accuracy of the four-digit HLA calls with sequencing-based ground truth sets from our Sierra Leone cohort, as well as Esan and Mende individuals from 1KGP. Imputation accuracies compared to the sequencing-based calls in Sierra Leone ranged from 89.2% to 97.6% (Fig. 4a). An additional 76 and 84 Mende and Esan individuals from our Sierra Leone and Nigeria cohorts, respectively, were typed for HLA genes *A*, *B*, *C*, *DQB1* and *DRB1* as part of 1KGP^53^. For these groups, imputation accuracy ranged from 91.4% to 99.2% (Fig. 4a). These comparisons showed adequate imputation of HLA alleles from SNP genotypes for our cohort.

We examined association of the four-digit HLA alleles with Lassa fever susceptibility phenotypes. No HLA alleles had a significant association with Lassa fever after correcting for multiple hypothesis testing (Fig. 4b). The allele with the strongest evidence of association considering both cohorts was DRB1*15:03, which had a *P* value of 0.089 in the Nigeria cohort and 0.064 in the Sierra Leone cohort, resulting in a meta-analysis *P* value of 0.013. B*15:16 and C*14:02 yielded *P* values of 0.0124 and 0.0259 in the Nigeria cohort, and DPA1*02:01 yielded a *P* value of 0.027 in the Sierra Leone cohort. After correcting for multiple hypothesis testing over all HLA tests, the most significant meta-analysis *q* value was 0.587 (Fig. 4b). Similarly, we did not find any associations for fatal outcomes after correcting for multiple hypothesis testing (*q* < 0.05). We tested the 41 novel HLA alleles that were discovered in our Sierra Leone cohort in a similar analysis (Methods), but none were significant.

## Discussion

Over a 10 year period we completed the first GWAS of infection with a risk group 4 pathogen reported to date. Our cohorts were recruited in remote parts of West Africa where Lassa fever is most prevalent. They reflected the paradoxical clinical heterogeneity of Lassa fever, with high fatality rates among people with LASV and high LASV seroprevalence among population controls. We find that an intronic variant within *GRM7* and a variant downstream of *LIF* are significantly associated with Lassa fever in the Nigeria cohorts and meta-analysis of the two cohorts, respectively. We identified candidate variants that approach, but do not reach, genome-wide significance in susceptibility analyses.

Several of the loci identified in our study contain genes with potential connections to Lassa fever biology. *LIF* encodes an interleukin 6 family cytokine that was previously shown to protect against lung injury in mouse models of respiratory syncytial virus infection^54^ and to be up-regulated in acute HIV infection^55^ and meningococcemia^56^. Altered regulation of this pleiotropic cytokine due to host variation could impact Lassa fever severity, giving rise to the observed association with fatality. *GRM7* may function in viral entry akin to *GRM2* in coronavirus disease 2019 or could be involved in immune activation as was seen in a recent knock-out model of anaphylaxis^46^. In addition, *GRM7* plays an important role in maintenance of hearing by inner-ear hair cells^47^; interestingly, hearing loss is a notable symptom of Lassa fever^48^. MPRA of the significant GWAS loci pinpointed the specific variants most likely to exert regulatory effects in the genome. None of these variants co-localized with expression quantitative trait loci in the Genotype-Tissue Expression dataset, but this might reflect the relative lack of African ancestry individuals in this resource^57^.

The variants reported here have ORs ranging from 6.87 to 9.19 for the susceptibility GWAS and as high as 15.4 for the outcome analyses (Tables 1 and 2). Intriguingly, the associated risk alleles are mostly uncommon, ranging from 1% to 5% frequency in our cohorts. Given their low frequency, they might be expected to have larger biological effects than what is typically seen for common variants^58^. Furthermore, the low allele frequency may reflect strong purifying selection, with the ubiquitous virus and high CFR purifying the risk allele from the population. Alternatively, the large effect sizes might reflect ‘winner’s curse’, in which only reporting variants that pass, or approach, genome-wide significance results in systematic upward bias of reported effect sizes in GWAS^59^. Larger replication studies and further biological characterization will be needed to clarify these signals.

We used our data to test a hypothesis that positive selection for genetic variation at the *LARGE1* locus provides protection from Lassa fever^6,16,17^. We found that a haplotype with long-range LD, indicative of recent positive selection, is nominally associated with reduced likelihood of Lassa fever in the Nigeria cohort but not in the Sierra Leone cohort. We reported promising support for this hypothesis in the 2011–2014 cohort, but this did not replicate in the subsequent recruitment from 2016–2018 (Extended Data Fig. 5). The discrepancy between cohorts might represent false positives in the first, power-limited, study or underlying differences between these temporally separated cohorts. It is noteworthy that, after the Ebola outbreak from 2013 to 2016, the number of suspected cases at Irrua Specialist Teaching Hospital (ISTH) surged^24^. Genetic epidemiology did not find evidence that a particular viral variant or extensive human-to-human transmission underpinned the surge, suggesting that it may have been driven by increased surveillance. Larger cohorts and deeper phenotypic characterization will be required to evaluate the hypothesis of *LARGE1* mediated genetic resistance to Lassa fever susceptibility.

We faced four major obstacles that will inform the design of similar studies: small sample sizes, uncertainty in case and control definitions, impact of environmental variables and insufficient characterization of genetic diversity in African populations.

Achieving large sample sizes for human studies of BSL-4 pathogens is challenging. Very few cases are documented annually, for example, less than 1,000 in Nigeria, the most populous country in the LASV endemic region^10^. Lassa fever is prevalent in rural areas that are far from diagnostic centres, further hampering recruitment^60^. Few facilities have diagnostic capacity for LASV infection, and field-deployable LASV tests are not widely available. Therefore, only a fraction of Lassa fever cases are identified, most likely those in which extreme disease presentations motivated the patient to seek medical attention. Some practical investments that would help increase the detection and treatment of LASV infection include diagnostic centres in rural areas, field-deployable, point-of-care diagnostics, and integrated health systems.

Defining Lassa fever cases and controls remains difficult, owing to insufficient diagnostic assays and LASV’s genetic diversity. These factors may result in false negatives as well as false positives that reduce power. We mitigated these limitations by using viral sequencing to supplement diagnosis at both sites. Our study also relied on population controls with unknown prior exposure to LASV. We used serology to characterize prior exposure but could not test every control in our cohort. Furthermore, interpretation of serology data is challenging as asymptomatic infections may not lead to sustained seropositivity (leading to false negatives) or could reflect the presence of undocumented Lassa fever in the past rather than asymptomatic illness. In any of these scenarios, the controls would be expected to carry the same susceptibility alleles as the people with LASV, reducing power to detect associations. Questionnaires to elicit detailed disease histories coupled with deeper serological characterization may help to distinguish individuals with previous Lassa fever from those with asymptomatic infection.

Viral genetic diversity, previous infections and co-infections, patient comorbidities and other health factors can further reduce GWAS power. LASV has up to 27% nucleotide diversity such that the specific infecting viral sequence could greatly impact outcomes. Moreover, the lineages in Nigeria and Sierra Leone are so divergent that they could potentially have different mechanisms of interaction with the host. In addition, previous infections with other endemic pathogens or co-infections with other pathogens could be a driver of observed symptoms and disease outcomes^61^. In future studies, metagenomic sequencing could define the genome of the infecting LASV strain while identifying the presence of co-infections, allowing these factors to be accounted for in the association model.

African populations are genetically diverse, with low levels of LD, and are under-studied, posing a challenge to GWAS of infectious diseases present mainly in Africa^62^. This issue was directly illustrated in our study; our relatively small HLA sequencing cohort of 297 individuals nevertheless identified 41 novel alleles. GWAS relies on imputing causal variants based on a relatively small number of variants included on the genotyping array. Accurate imputation requires the existence of genotyping arrays containing representative variation from the population of interest and large whole-genome sequencing reference panels, both of which are deficient for African populations. Reduced imputation accuracy can dramatically reduce power, making studies such as this one more challenging. Continuing efforts to improve our understanding of genetic variation in African populations will allow further insights into potential links between genetics and disease.

In summary, our work paves the way for follow-up studies on Lassa fever and other group 4 microbial pathogens and has contributed to an improved genetic data resource for African populations.

## Methods

### Institutional review board ethical review and approval

This work was approved by the following institutional review boards and local ethics committees: Nigerian National Health Research Ethics Committee and ISTH (ISTH/HREC/20170915/22), Sierra Leone Ethics and Scientific Review Committee (070716), Tulane University Human Research Protections Office (10-191330) and Harvard University Area Committee on the Use of Human Subjects (19-0023). Enrolment procedures and sampling efforts were carried out at Irrua Specialist Teaching Hospital (ISTH), Kenema Government Hospital (KGH) (IRB 070716) and their surrounding communities with participant consent or through a waiver of consent granted by the appropriate institutional review board/local ethics committee. Some samples shared with the study collaboration include those stored at the respective hospitals as clinical excess or approved for secondary use.

### Lassa fever case definition and recruitment

#### ISTH, Nigeria

We recruited people with Lassa fever at ISTH between 2011 and 2014 and between 2016 and 2018 with a gap from 2014 to 2016 due to the Ebola outbreak in West Africa that temporarily halted research operations. We performed molecular diagnostic testing for all individuals suspected to have LASV who met clinical diagnostic criteria for Lassa fever including fever >38 °C for less than 3 weeks, absence of signs of local inflammation, absence of clinical response to anti-malarials and additional major and minor signs^63^. Individuals suspected to have LASV who were positive by molecular diagnostic testing were recruited to the study following informed consent.

#### KGH, Sierra Leone

People with Lassa fever were recruited at KGH between 2011 and 2018 with a gap from 2015 to 2016 due to the Ebola outbreak in West Africa. Individuals suspected to have LASV included those who met clinical diagnostic criteria for Lassa fever^63^ and were positive by either ELISA for a LASV antigen or immunoglobulin M antibody against LASV^25,64^. We performed virus sequencing from a subset of enrolled people with LASV^12^. We only included data from individuals suspected to have LASV who were either antigen-ELISA positive or viral sequencing positive with reads per kilobase million of >1 in the GWAS.

#### Population control recruitment

Study staff at ISTH and KGH recruited population controls through outreach efforts to villages with a recent history of Lassa fever cases. Village controls (Supplementary Table 2) were healthy individuals who were recruited from the same household and/or village as people with LASV, prioritizing unrelated individuals where possible. Trio controls (Supplementary Table 2) were healthy families of mother, father and child from the Esan population in Nigeria and the Mende population in Sierra Leone who were recruited jointly with phase 3 of the 1KGP^51^. The informed consent criteria for this project were developed by the Samples and Ethical, Legal and Social Implications Group of the National Human Genome Research Institute^51^ and extends to the analyses we carried out in this study.

See Supplementary Note for more details about real-time quantitative PCR, sequencing and ELISA assays.

### DNA extraction and genotyping

For all consenting study participants, we extracted buffy coats from the diagnostic blood draw after they were spun at 1,500 *g* for 10 min. We collected the buffy coat into a 1.5 ml tube, extracted DNA using the Qiagen DNAeasy kit following manufacturer’s instructions and shipped DNA samples to the Broad Institute.

For samples collected between 2011 and 2014, genotyping was performed at the Broad Institute’s Genomics Platform on either the Infinium Omni 2.5 M or the Omni 5 M arrays. For samples collected after 2015, genotyping was performed at Illumina in San Diego on the H3Africa array.

### Variant preprocessing and genome-wide association

See Supplementary Note for detailed description of variant preprocessing, principal component analyses, GWAS analysis and meta-analysis. Briefly, we first filtered variants that showed significantly different calls across genotyping arrays. We then merged the remaining samples into a single VCF file and ran imputation using the Sanger Imputation Service^65^ and EAGLE2 v2.0.5 for phasing^66^ using the African Genome Resources reference panel.

We conducted all genetic association tests using mixed models logistic regression as implemented in version 1.2.0 of SAIGE^34^ using the leave-one-chromosome-out option. We used genotyped variants that passed quality control filters to compute PCs and the genetic relatedness matrix. We used sex, array (H3Africa versus Infinium Omni) and PCs as covariates. We used METAL (version corresponding to 25 March 2011 release)^67^ to meta-analyse the results of the Nigeria and Sierra Leone cohorts using the default option of weighting each cohort by sample size.

### MPRA

See Supplementary Note for details on MPRA methods.

### *LARGE1* haplotype analysis

To define the LARGE-LRH, we extracted phased imputed genotype data from our cohort for the region on chromosome 22 between base pairs 33,870,000 and 34,470,000 in GRCh37, which corresponds to the previously defined region of the haplotype^17^. We then filtered out variants with minor allele frequency below 0.05 and clustered the corresponding haplotypes using *K*-means as implemented in Scikit-learn version 0.21.3 with *K* = 2. We identified individuals who were homozygous (coded as 2), heterozygous (coded as 1) or had 0 copies of the haplotype (coded as 0) and tested for association with Lassa fever phenotypes using SAIGE as described above and in the Supplementary Note.

To tag individuals from the 1KGP dataset who were carrying the LARGE-LRH, we identified the five SNPs that were most correlated with the clustering-defined haplotype in our dataset based on Pearson correlation. These were rs59015613, rs16993014, rs4525791, rs8135517 and rs59594190, all of which had a Pearson correlation >0.92 with the LARGE-LRH. We then used the phased 1KGP data to label haplotypes as the LARGE-LRH if three or more of the linked tag SNPs were present. The results were unchanged if we required only 2 or more linked SNPs to be present, and requiring 5/5 tag SNPs to be present only decreased the number of called haplotypes called from 252 to 250.

### HLA sequencing, imputation and association analysis

#### Sequencing-based HLA typing

We performed sequencing-based HLA typing on samples from 297 Sierra Leone study participants. We generated sequencing libraries with the TruSight HLA v2 Sequencing Panel, following manufacturer’s instructions, and sequenced the samples on Illumina Miseq instruments at either the Broad Institute, Boston, MA, or Scripps Institute, La Jolla, CA. We assigned HLA calls from the raw sequencing reads using the Assign 2.0 TruSight HLA Analysis Software.

#### HLA imputation

We developed an HLA imputation panel from 3,608 African Americans^68^. This consisted of sequencing-based HLA calls for the *HLA-A*, *HLA-B*, *HLA-C*, *HLA-DPA1*, *HLA-DPB1*, *HLA-DQA1*, *HLA-DQB1* and *HLA-DRB1* genes, as well as SNP genotyping data from either the Affymetric Genome-Wide Human SNP Array 6.0 (2259) or the Infinium Omni 2.5 M array (1349). We imputed SNPs on chromosome 6 for these individuals using the same pipeline as for our GWAS cohort (Sanger Imputation Service with Eagle2 phasing and the African Genome Resources panel). We then subsetted to the HLA region (GRCh37 position between 28191116 and 34554976) and used the HIBAG version 1.22 software hlaParallelAttrBagging function to create an HLA reference index consisting of seven independent classifiers that could be used to predict HLA from imputed SNP inputs^69^. We then used those indices with HIBAG’s hlaPredict function to impute HLA types for our cohort.

We evaluated imputation accuracy against the sequence-based typing ground truth sets by calculating the percentage of alleles called correctly out of 2*N* where *N* is the total number of individuals in the ground-truth set. We excluded novel alleles from these calculations for the Sierra Leone set. We also estimated the accuracy of our imputation for HLA-A, HLA-B, HLA-C, HLA-DQB1 and HLA-DRB1 for separate dataset of 76 Mende and 84 Esan individuals from the 1KGP who were genotyped in our cohort and HLA-typed by Gourraud et al.^53^.

#### HLA association analysis

We calculated dosages for each allele by summing the posterior probabilities for each genotype output by HIBAG that contained the allele. We only included alleles with minor allele frequency above 1% in a cohort for association analysis. We then used the same mixed logistic regression model as for the SNP-based GWAS to associate the HLA alleles with Lassa fever phenotypes, using the dosage for each allele as the predictor and using sex and PCs as fixed effect covariates.

### Reporting summary

Further information on research design is available in the Nature Portfolio Reporting Summary linked to this article.

### Supplementary information

Supplementary Information.

Reporting Summary

Supplementary Table 1 *P* values and estimated meta-analysis *z*-scores for the susceptibility GWAS.

Supplementary Table 2 *P* values and estimated meta-analysis *z*-scores for the outcome GWAS.

Supplementary Table 3 MPRA results data for the K562 cell line for the lead GWAS association peaks.

Supplementary Table 4 MPRA results data for the HepG2 cell line for the lead GWAS association peaks.

Supplementary Table 5 Analogous MPRA data for the LARGE1 long-range haplotype.

## Figures and Tables

**Extended Data Table 1:**
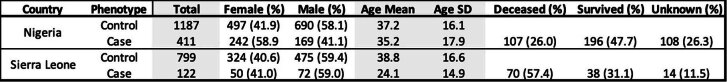
Summary of GWAS collections for the Nigerian and Sierra Leonean cohorts

Includes breakdown of samples by sex, includes age mean and SD for each sample set, and breakdown of clinical outcome for cases.

**Extended Data Table 2:**
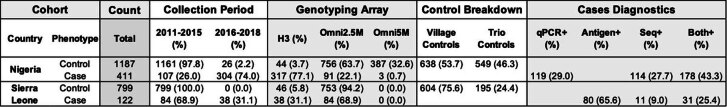
Detailed summary of GWAS collections

Includes breakdown of samples by collection time period, genotyping array, split of controls into village and trio recruitments, and diagnostic categories of cases. For the case diagnostic category, Nigerian cases were positive by RT-qPCR (qPCR+) and/or sequencing (Seq+), whereas Sierra Leonean cases were positive by antigen ELISA (Antigen+) and/or sequencing. The last column Both+ specifies the number of cases who were positive by both sequencing and RT-qPCR or ELISA.

**Extended Data Table 3:**
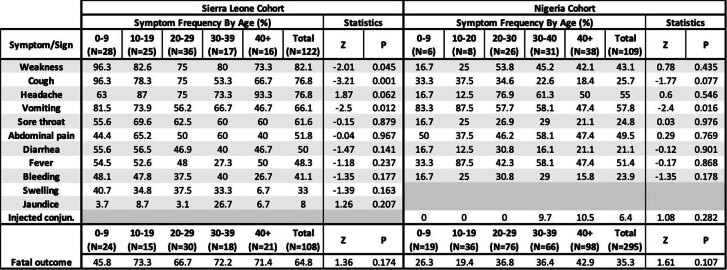
Overview of clinical symptoms

Percentage of cases with a clinical sign or symptom at the time of admission, stratified by age. Below each age range is the number of individuals in that group with clinical data available. We report the large-sample approximation test statistic (Z) and P-value (P) for a Wilcoxon Ranksum test comparing the median age of subjects with and without each symptom. Conjunctival injection was recorded for the NG cohort but not the SL cohort, and lower extremity swelling or jaundice were recorded for the SL cohort but not the NG cohort. Bleeding includes any observed bleeding such as epistaxis, hematemesis, hematuria, melena, and hematochezia. Fever is defined as a temperature on admission of greater than 37.8 degrees celsius.

**Extended Data Table 4:**
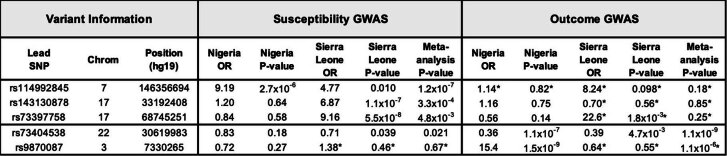
Comparison of lead variants between the outcome and susceptibility GWAS analyses

Displays odds ratios (OR) and P-values for lead variants in either the susceptibility GWAS (top) or outcome GWAS (bottom). P-values are based on saddlepoint-approximated score tests (SAIGE), while meta-analysis P-values are derived from meta-analysis (METAL) of P-values generated from each cohort. Odds ratios are computed from Firth logistic regression. *Variants with an asterisk were excluded from the corresponding analysis due to quality control filters but are included here for completeness.

**Extended Data Table 5:**
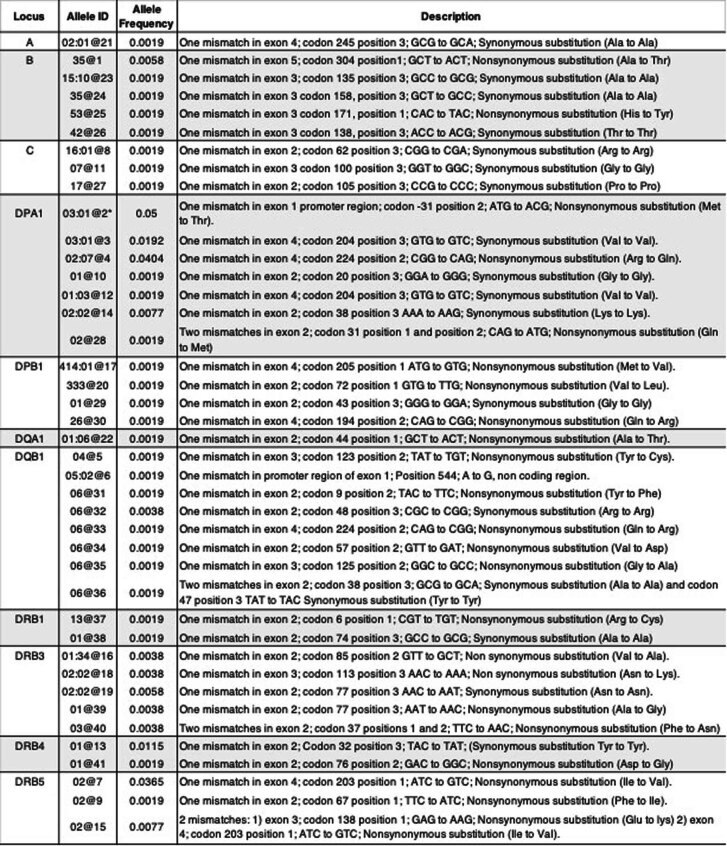
Uncovered HLA alleles

Novel HLA alleles identified in sequence-based HLA typing of 297 Sierra Leoneans.

## Data Availability

Raw de-identified genetic data from this study have been submitted to the European Genome–Phenome Archive (dataset IDs EGAD00010002510 and EGAD00010002509). The vcf file containing these data can be accessed by registering an account with EGA (https://ega-archive.org/register/) and making a request to the Data Access Committee, following which a download will be made available to the account holder. Summary statistics for genetic analyses reported in this study are available in the GWAS catalogue (https://www.ebi.ac.uk/gwas/) under accession codes GCST90301246, GCST90301247, GCST90301248 and GCST90301249. Meta-analyses of the GWASs are available in Supplementary Tables 1 and 2. Summary statistics for the MPRAs are included in Supplementary Tables 3 and 5. Data from the 1KGP are available at https://www.internationalgenome.org/data/. Genome assembly hg19 is available at https://www.ncbi.nlm.nih.gov/datasets/genome/GCF_000001405.13/.

## Code availability

Data analysis scripts employed in this manuscript are publicly available on GitHub at https://github.com/dylkot/lassa_fever_gwas.
