# Supplementary material for: Genome-wide association study identifies human genetic variants associated with fatal outcome from Lassa fever
Source: Nat Microbiol. 2024 Feb 7;9(3):751–62. doi: 10.1038/s41564-023-01589-3 (PMC10914620; doi:10.1038/s41564-023-01589-3)
Supplement: Supplementary file 1 — Supplementary Information. [file 41564_2023_1589_MOESM1_ESM.pdf]

nature microbiology

Article

https://doi.org/10.1038/s41564-023-01589-3

Genome-wide association study identifies human genetic variants associated with fatal outcome from Lassa fever

In the format provided by the authors and unedited

Supplementary Note

Diagnostic Testing

Blood Draws

5-10mL blood draws were collected from suspected cases in an EDTA tube. This was spun at 1500g for 10 minutes to collect plasma for diagnostic testing. Plasma was either inactivated with AVL for RNA-detection via RT-qPCR or viral genome sequencing, or processed directly for antigen or antibody detection ELISA.

Rt-qPCR

From 2011 to 2014, ISTH study staff performed RT-PCR targeting the *GPC* gene[72] as the primary diagnostic and positive cases were recruited into the study. However, due to concerns about false positives of this initial assay, a confirmatory RT-qPCR assay was performed at the Broad institute in Boston using primers against the LASV S segment (forward: CCCAAGCYCTHCCYACAAT, reverse: AACCCTTATGAGAAYATACTBTAYAA) and a subset of patients underwent next-generation viral sequencing[12]. We only included data from recruited cases who were positive by this latter RT-qPCR or who had positive LASV sequencing with greater than 1 viral reads per kilobase (RPKM) in the GWAS analysis.

Between 2016-2018, ISTH patients who met clinical diagnostic criteria for LF were tested at ISTH with 2 RT-qPCR assays, one targeting the *GPC* gene (RealStar LASV RT-PCR Kit 1.0 CE, Altona Diagnostics, Hamburg, Germany) and a second targeting the LASV L segment [73-74]. Suspected cases from this period who were positive by either RT-qPCR assay were recruited to the study following informed consent. A subset of these cases also underwent viral sequencing[24]. We only included suspected LF cases who were positive by both of the RT-qPCR assays, or by viral genomic sequencing (with > 1 RPKM from the viral genome) in the GWAS.

Viral Sequencing

We performed next-generation viral sequencing for a subset of recruited cases from Nigeria and Sierra Leone following protocols described in detail in Matranga Et al., 2016[73]. Library preparation and sequencing occurred at the Broad Institute and Redeemer's University and this data is described in detail in (Andersen Et. al., 2015)[12] and (Siddle Et al., 2018)[24] (NCBI BioProject PRJNA254017 and PRJNA436552). Samples were sequenced and data was processed as described in those publications and cases with >1 RPKM (read per kilobase of transcript, per million mapped reads) of LASV were included as cases (Table S1).

ELISA

The ReLASV Ag ELISA and ReLASV IgM and IgG ELISAs were used to detect LASV antigen as well as anti-LASV IgM and IgG antibodies[25]. Antigen and antibody ELISAs were run on a routine basis for suspected cases at KGH. In addition, we also used the ReLASV IgM and IgG assay to test a subset of population controls recruited in Nigeria and Sierra Leone from 2011-2014 for antibodies.

In brief, the ReLASV Ag ELISA included LASV NP-specific rabbit polyclonal antibody and the ReLASV IgM and IgG ELISA are a mixture of ReLASV NP, glycoprotein complex (GPC), and Z matrix protein. Plasma was diluted 1:10, incubated for 60 minutes at 37 °C (ReLASV Ag) or 30 minutes at room temperature (ReLASV IgM and IgG), washed four times with 300 µL/well of a PBS-Tween. Peroxidase labeled LASV NP-specific rabbit polyclonal reagent was added and then incubated at room temperature for 30 minutes, and washed four times with 300 µL/well of PBS-Tween. Substrate (Moss, Inc. Pasadena, MD) was added (100 uL/well) and incubated for 10 minutes followed by stop solution. Samples were read at 450 nm with 650 nm subtraction with an OD450 nm cut-off of 0.09 (ReLASV Ag) or 450 nm with 650 nm subtraction (ReLASV IgG/IgM). The ReLASV IgM, IgG assays negative cut-off is OD = 0.226 with an intermediate cut-off of OD = 0.452 and OD = 0.170 with an intermediate cut-off of OD = 0.340, respectively.

Variant preprocessing and genome-wide association

Genotype and HLA calling

We used Illumina GenomeStudio version 2.0 to call genotypes from the raw array images. We used Illumina Assign 2.0 TruSight HLA Analysis Software to call HLA alleles from long read sequencing data.

Batch effect correction and variant imputation

We filtered any samples with overall missingness rate greater than 95% or with a genotype-based sex prediction that didn't match the expected sex.

We then combined genotype data from the H3Africa, Infinium Omni 2.5M, and Infinium Omni 5M arrays and kept 1,470,760 variants that were present on all 3 platforms and had a combined variant call missingness rate less than 10%. We subsequently filtered out any variants that had evidence of systematic differences between (1) two genotyping batches of samples typed on the H3Africa array, (2) samples typed on two batches of Infinium Omni 2.5M arrays with distinct probesets (HumanOmni2.5M-8v1_A vs HumanOmni2.5M-8v1-1_b), (3) samples types on Infinium Omni 2.5M and Infinium Omni 5M arrays, and (4) samples genotyped on the H3Africa array vs. the Infinium arrays.

First, we considered samples that were genotyped in more than one of the above groups and filtered variants that had >10% discrepancy in genotype calls between batches across all replicates. Second, we used the GMMAT package[75] to run mixed logistic regression analysis to identify variants with statistically significant differences in allele frequency between groups (see below for details on regression models). We ran this regression for samples from both Nigeria and Sierra Leone cohorts combined, using country and case-control status as covariates, as well as for the Nigeria and Sierra Leone cohorts separately using only case-control status as a covariate. We computed FDR-corrected p-values using the Benjamini-Hochberg method and excluded variants with a q-value below 0.1 in any of the above analyses. In total, this excluded 25,327 variants due to batch effect. We additionally filtered 5700 variants due to Hardy Weinberg equilibrium P-values that were less than $1x10^{-10}$.

Next, we imputed non-genotyped variants for this cohort using the Sanger Imputation Service[76] using EAGLE2 for phasing[77] and the African Genome Resources reference panel, which contains genomes from 4,956 individuals, almost entirely of African ancestry (https://imputation.sanger.ac.uk/?about=1). Excluding variants with imputation INFO score < 0.80, minor allele frequency < 0.01, genotype missingness rate less than 0.05, or Hardy Weinberg equilibrium P-value < $1x10^{-6}$, we obtained a final set of 12,783,971 and 12,522,562 variants tested in the primary susceptibility GWAS for the Nigeria and Sierra Leone cohorts respectively.

Principal component analysis (PCA)

Principal components (PCs) were used as fixed effects in the GWAS analysis. However, given the substantial levels of relatedness in the cohort, naively applying Principal Components Analysis (PCA) yielded PCs that were strongly driven by closely related individuals in the dataset (data not shown). To obtain principal components reflecting more distant relatedness, such as tribal ancestry, we therefore first identified closely related individuals in the dataset using the --make-grm-bin function in Plink2 on genotyped (non-imputed) variants[76,77] (https://www.cog-genomics.org/plink/2.0/). We then filtered individuals with relatedness coefficient > 0.05 using the --rel-cutoff function in Plink2 and ran PCA on the genotyped variants of the unrelated samples using the --pca 20 biallelic-var-wts function in Plink 2.0. Finally, we projected genomic data from all individuals onto these unrelated PCs using the --score command in Plink 2.0 with the no-mean-imputation variance-normalize flags. We visualized Skree plots showing the variance explained for each PC. Based on the apparent elbow in these plots, we selected 6 PCs for the susceptibility analyses and 4 PCs for the outcome analyses for both Nigeria and Sierra Leone.

Genome-wide association analysis and meta-analysis

We conducted all genetic association tests using mixed models logistic regression as implemented in version 1.2.0 of SAIGE[35]. We first filtered any samples that were not cases or controls for the given phenotype, as well as any sample replicates, keeping the replicate with the lowest genotype missingness rate. We then used genotyped variants

that passed quality control filters to compute principal components as described above. We then fit the null model using these same genotyped variants with the step1_fitNULLGLMM.R script provided with the SAIGE package. We used sex, array (H3 Africa vs. Infinium Omni), and PCs as covariates. We then scored the effects of imputed variants that passed quality control filters using imputed dosage values as the predictors with the step2_SPAtests.R script. We used the LOCO=True, minMac=20, --is_Firth_beta=TRUE, and --pCutoffforFirth=0.01 options to use a relatedness matrix excluding the chromosome of the variants being tested, excluding variants with fewer than 20 minor allele counts, and computing effect sizes using Firth logistic regression for variants with p-value less than 0.01. We used METAL[68] to meta-analyze the results of the Nigeria and Sierra Leone cohorts using the default option of weighting each cohort by sample size.

LARGE1 Massively Parallel Reporter Assay (MPRA)

We performed an MPRA following previously described methods[51] with modifications described below.

MPRA variant selection

We identified 3,417 variants including SNPs, insertions, and deletions overlapping the LARGE haplotype region (between chr22 33600759 and 34499558 in hg19) that had minor allele frequency greater than 5%. From these we selected a final set of 1,674 variants that were linked to the LARGE1 haplotype with an absolute value Pearson correlation greater than 0.15. From these variants, we constructed 5,860 200 bp oligonucleotides, containing either the reference or alternate alleles for each variant and it's flanking genomic sequence with the allele centered in the middle of the oligo. When multiple (2-4) variants overlapped the genomic sequence contained within an oligo sequence, we created oligos for all combinations of the reference and alternate alleles.

MPRA vector assembly

200bp oligos were synthesized by Agilent including 15 base pairs of adapter sequence at both ends (5'ACTGGCCGCTTGACG, CACTGCGGCTCCTGC3'). After synthesis, adapters and 20 bp barcodes were attached via 12X 50 µL PCR reactions using the NEBNext Ultra II Q5 Master Mix (NEB, M0544L) with primers MPRA_v3_F (10 µM) and MPRA_v3_R (10 µM) and the following cycle conditions: 98°C for 20 seconds, 15 cycles (98°C for 10 sec, 60°C for 15 sec, 72°C for 45 sec), 72°C for 5 minutes. The product was then subject to two 1X AMPure SPRIs (Beckman Coulter, A63881) and eluted in 200 µL water. pGL4:23:∆xba∆luc was then digested by SFiI (NEB, R0123S) at 50°C for one hour. The resulting digested backbone and oligo product were then assembled via

Gibson assembly reaction (NEB, E2611L) using 1 μg digested plasmid and 1 μg oligos and incubation at 50°C for one hour and purified by a 1.2X AMPure SPRI and eluted in 20 μL. 10 μL of the assembled construct was then electroporated (2kV, 200 ohm, 25 μF) into 100 μL 10-beta e.coli (NEB, C3020K). Electroporated cells were split into 8 tubes and grown in 2 mL SOC prior for one hour at 37°C. Subsequently, the 8 aliquots were independently expanded in 20 mL LB supplemented with 100 μg/mL of carbenicillin for 6.5 hours at 37°C. Afterwards, bacteria were pooled and the resulting plasmid purified via QIAGEN Plasmid Plus Maxi Kit (Qiagen, 12963). Serial dilutions estimated the combined complexity being ~1.7x10^8 CFU.

20 μg of the resulting vector was then cut with 200 units of AsiSI (NEB, R0630L) and 1x CutSmart buffer in a 500 μL reaction at 37°C for 3.75 hours followed by a 1.5X AMPure SPRI cleanup. The linearized vector and an amplicon containing a minimal promoter, GFP open reading frame and partial 3'UTR was then assembled together via a Gibson reaction using 10 μg of the AsiSI linearized vector and 33 μg of the GFP amplicon in a 400 μL reaction at 50°C for 1.5 hours followed by heat inactivation for 20 minutes at 80°C. The entire reaction was cleaned by a 1.5X AMPure SPRI and eluted in 55 μL. The elution from the cleanup was then digested again to remove any uncut plasmids with 50 units of AsiSI, 5 units of RecBCD (NEB, M0345S), 10 μg of BSA, 0.1 mM of ATP, and 1X NEB Buffer 4 in a 100 μL reaction for 1 hour 40 minutes at 37°C. Subsequently, 9 μL of 10 mM ATP was added to the 100 μL reaction and the digestion continued at 37°C for 4 hour 20 minutes (6 hours total) followed by heat inactivation for 20 minutes at 80°C and a SPRI purification.

The vector library was generated by electroporating 50 μL 10-beta e.coli with 5 μL DNA (2kV, 200 ohm, 25 μF). Bacteria was split into 3 separate tubes, each with 2 mL SOC and grown for 1 hour. After the 1 hour recovery, all 3 tubes from each batch were combined into 200ml of LB with 100 μg/mL of carbenicillin and grown for 9 hours. The plasmid was then prepped via the Qiagen Maxiprep kit.

We synthesized the LARGE promoter (capitalized letters) within multicloning sites in the puc57 vector from genscript:

ctagcctcgaggatatcaagatctggcctcggcggccGAAGCCGGCGCATCTCGGAGGCGGCGGC
GGCGGCCAAGGCCGGCGAGCGCTCCCGGCGGCGGGGGCCGCCCGCCTCGGCT
CCCCGCACCCACCGCGCCGCGATCCACTCGCCGCGCCTCCGCTCCCGTGACCTT
CCCGGGGCGCCTCCCCTAGCCCCGCGCCCCCGGCCCCGCGCCCCAGGCCGGGG
CGAGGCCTTTTCCGGCGCTTCTTTCCCGCGGAGCCGCGGGCGGGCGGCGCAGG
CCCTGGGGGAGAGCGCGCCGCGGCCGGTTGCAGCCCCCCCCGCGCCGCCGCGT
TCGGCGCCCGGCCCGGCCAGTCTGCTCCTGCCCCGCCGCCGCGCCGGAGCCCG
GGCGCCCGAAGCTGGGGGCGCGGCCGCGCTCGTCTCGCCGGGCTGTTCCATGgt
gagcaagggcgag

We cloned this sequence using an EcoRV, Nco1 (NEB) double digest to extract the promoter from puc57, and insert it into the MPRA vector replacing the minimal promoter using 2x quick ligase (NEB).

The final vector library was generated by electroporating 4 batches of 100 µL 10-beta e.coli with 10 µL DNA (2kV, 200 ohm, 25 µF). Each batch of bacteria was split into 3 separate tubes, each with 2 mL SOC and grown for 1 hour (twelve tubes in total across all 4 batches). After the 1 hour recovery, all 3 tubes from each batch were combined into 1.5 L of LB with 100 µg/mL of carbenicillin in a single 2.8 L flask and subsequently grown for 9 hours (four 2.8 L flasks with 1.5 L LB across all 4 batches). The plasmid was then prepped via the Qiagen Gigaprep kit (Qiagen, 12191).

Transfection

GM12878s (Coriell) were cultured in RPMI (Thermo Fisher, 61870036) containing 15% FBS and 1% 10X Penicillin-Streptomycin (Pen-Strep; Thermo Fisher, 15140122; Corning, 30-002-CI). Five total replicates, grown on different days to ~1 million cells per mL, were transfected. Per replicate transfection, 150 million cells were pelleted at 300 x g and resuspended in 1.2 mL RPMI containing 150 µg of the MPRA library. Cells were electroporated using the Neon transfection system and the setting of 3 pulses of 1200 V, 20 ms with the 100 µL kit (Thermo Fisher, MPK10096). After transfection, each replicate was recovered for 48 hours in 150 mL RPMI containing 15% FBS without Pen-Strep. After the first 24 hours of recovery, cells were split 1:2 to avoid overgrowth. After 48 hours of recovery, the cells were pelleted via centrifugation, PBS-washed once, flash-frozen using liquid nitrogen, and then stored at -80°C.

Transfection efficiency was assessed by checking GFP fluorescence from test transfections using a control vector containing GFP. We required a minimum of 50% of live cells fluoresced after transfection.

RNA isolation and MPRA RNA library generation

Frozen cell samples were processed following the MPRA protocol in (19). Briefly, RNA was extracted from the Qiagen Maxi RNeasy kit (Qiagen, 75162), without the on-column DNase digest. A DNase reaction was then performed to remove remaining MPRA library vectors. The GFP in the total RNA was then captured via a hybridization reaction using streptavidin beads (ThermoFisher Scientific, 65001) and a mixture of 3 GFP RNA-targeted biotinylated oligos (table S5). A second DNase reaction was then performed to remove any undigested library vectors. Following a RNA SPRI (Beckman Coulter, A63987) cleanup, the RNA was then converted to cDNA in a Superscript III (ThermoFisher Scientific, 18080044) reaction using MPRA_v3_Amp2Sc_R (table S5). The cDNA was then cleaned via AMPure SPRI and the relative cDNA abundance across all cell type samples and MPRA library vector was estimated via qPCR by comparing their cycle thresholds (number of cycles required to amplify above background). In total, we had 4 replicates per cell type. All cell type replicates (with the

exception of NPC samples which were processed later) were normalized to approximately the same concentration and cycled for 10 cycles in a PCR reaction using NEBNext Ultra (NEB, M0544L) to amplify the cDNA using the primers MPRA_v3_Illumina_GFP_F and TruSeq_Universal_Adapter (table S5). Five MPRA plasmid library replicates, input normalized to achieve the same PCR output abundance, were amplified for 10 cycles. Due to the lower amount of GFP RNA output from our NPC samples, we used approximately 3 times lower RNA and cycled the NPC samples 2 cycles higher (12 cycles total). The resulting amplified products from all cell types was then subject to another round of PCR with 6 cycles to attach custom p7 and p5 Illumina adapters with unique sample indices (table S5).
We used the Agilent 2200 TapeStation (using the D1000 screentape reagents (Agilent, 5067-5585) to acquire molar estimates of final PCR products and pooled samples for subsequent sequencing. Samples were sequenced with a S4 flowcell (2 x 150 bp) on a NovaSeq using the sequencing service from the Broad Institute. For the NPC samples, we had sequenced them separately on a NextSeq using the NextSeq 500/550 High Output Kit v2.5 (20024906) (1 x 75 bp).

MPRA data processing and analysis
Data from the MPRA was analyzed as previously described[78] using MPRAmatch, MPRAcount and MPRAmodel (https://github.com/tewhey-lab/MPRA_oligo_barcode_pipeline and https://github.com/tewhey-lab/MPRAmodel). Briefly, barcode counts were normalized across replicates for each oligo. Significant differences between DNA plasmid count and RNA count were identified using a negative binomial generalized linear model. This analysis was performed for all data (single variants as well as variant pairs) and coefficients from the regression for activity, allele-specific activity, and interactions between pairs of variants were obtained as well as their standard errors from Wald tests. When an allele was tested against multiple genetic backgrounds because the oligo overlapped with other variants, we plot the most significant of the tested combinations in Figure 3C and describe the most significant allele combination in the text.

GWAS Lead Variant Massively Parallel Reporter Assay (MPRA)

The GWAS lead variant MPRA was performed as above but with additional modifications which we detail below.

MPRA variant selection

To select variants, we started from 39 SNPs that passed a significance threshold of $1x10^{-7}$ in the susceptibility or outcome GWAS for Nigeria or Sierra Leone, or in a meta-analysis of the two countries. We then identified a total of 234 SNPs to include in the MPRA that had a linkage disequilibrium R^2 of 0.5 or greater with any of the 39 lead SNPs.

MPRA vector assembly

230 bp oligos were synthesized as part of a larger 300K MPRA library by Twist Biosciences including 15 base pairs of adapter sequence at both ends (5’ACTGGCCGCTTGACG, CACTGCGGCTCCTGC3’). After synthesis, adapters, additional linker sequences, and a 20 bp barcodes were attached via 24X 50 µL PCR reactions using the NEBNext Ultra II Q5 Master Mix (NEB, M0544L) with primers MPRA_v3_F (10 µM) and MPRA_v3_20I_R (10 µM) and the following cycle conditions: 98°C for 20 seconds, 6 cycles (98°C for 10 sec, 60°C for 15 sec, 65°C for 45 sec), 72°C for 5 minutes. The product was then subject to one 0.8X AMPure SPRIs (Beckman Coulter, A63881) and eluted in 200 µL water. pMPRAv3:Δluc:ΔxbaI (addgene: 109035) was then digested by SfiI (NEB, R0123S) at 50°C overnight. The resulting digested backbone and oligo product were assembled via a Gibson assembly reaction (NEB, E2611L) using 2 µg digested plasmid and 2.2 µg oligos and incubation at 50°C for one hour and purified by a 1.2X AMPure SPRI and eluted in 20 µL. 1 µL of the assembled construct was then electroporated (2kV, 200 ohm, 25 µF) into 50 µL 10-beta e.coli (NEB, C3020K). Electroporated cells were split into 10 tubes and grown in 1 mL SOC prior for one hour at 37°C. Individual aliquots were independently expanded in 20 mL LB supplemented with 100 µg/mL of carbenicillin for 6.5 hours at 37°C and CFUs for each aliquot were estimated using serial dilutions. We selected 10 x 20 mL aliquots to achieve a target CFU of 86,000,000 (average of 286.6 barcodes per oligo), bacteria was pooled and the resulting plasmid purified via QIAGEN Plasmid Plus Maxi Kit (Qiagen, 12963) to generate the pMPRAv3:Δorf library.

To insert the reporter gene 10 µg of the pMPRAv3:Δorf library vector was cut using 100 units of AsiSI (NEB, R0630L) and 1x CutSmart buffer in a 400 µL reaction at 37°C overnight followed by 2 columns of NEB Monarch PCR + DNA Cleanup Kit (#T1030S). The linearized vector and an amplicon containing a minimal promoter, GFP open reading frame and partial 3’UTR was then assembled together via a Gibson reaction using 1.6 µg of the AsiSI linearized vector and 5.28 µg of the GFP amplicon in a 250 µL reaction at 50°C for 1.5 hours.. The entire reaction was cleaned by a 1.5X AMPure SPRI and eluted in 40 µL. The elution from the cleanup was then digested again to remove any uncut plasmids with 50 units of AsiSI, 5 units of RecBCD (NEB, M0345S),

10 μg of BSA, 1 mM of ATP, and 1X NEB Buffer 4 in a 100 μL reaction incubated at 37°C overnight followed but a 1.5x SPRI purification using a 40 uL elution volume.

The final MPRA plasmid library was generated by electroporating 2 batches of 350 μL 10-beta e.coli with 14 μL of pMPRAv3:oligo:minP:GFP gibson assembled DNA (2kV, 200 ohm, 25 μF). Each batch of bacteria was split into 6 separate tubes, each with 2 mL SOC and grown for 1 hour (twelve tubes in total across all 4 batches). After the 1 hour recovery, all 6 tubes from each batch were each combined into 0.5 L of LB with 100 μg/mL of carbenicillin in a single 2.8 L flask and subsequently grown for 16 hours at 30C (six 2.8 L flasks with 0.5 L LB across all 2 batches). The plasmid was then prepped via the Qiagen Gigaprep kit (Qiagen, 12191).

<u>Transfection</u>

Seven hundred million HepG2 or K562 cells were transfected using the Neon™ Transfection System 100ul Kit with 10ug of the MPRA library per ten million cells. Twenty-four hours after transfection cells were harvested, rinsed with PBS and collected by centrifugation. After adding RLT buffer (Rneasy Maxi kit), dithiothreitol and homogenization, cell pellets were frozen at -80°C. For each cell type 5 biological replicates were processed with no more than two replicates performed on the same day and these were performed using independently expanded batches of cells.

<u>RNA isolation and MPRA RNA library generation</u>

RNA was extracted from frozen cell homogenates using the Qiagen RNeasy Maxi kit. Following DNase treatment, a mixture of 3 GFP-specific biotinylated primers were used to capture GFP transcripts using Dynabeads™ MyOne™ Streptavidin C1 beads (Life Technologies) or Sera Mag Beads (Fisher Scientific). After another round of DNase treatment, complementary DNA was synthesized using SuperScript™ III (Life Technologies) and GFP mRNA abundance was quantified by qPCR to determine the cycle at which linear amplification begins for each replicate. Replicates were diluted to approximately the same concentration based on the qPCR results, and first round PCR (6-13 cycles) with primers #781 or 801 and #782 or 802 (Supplementary Table 1) was used to amplify barcodes associated with GFP mRNA sequences for each replicate. A second round of PCR (6 cycles) was used to add Illumina sequencing adaptors to the replicates. The resulting MPRA barcode libraries were spiked with 0.01-1% PhiX and sequenced using Illumina single-end 20 bp chemistry (with dual 8 bp i5 and i7 index reads).

<u>MPRA data processing and analysis</u>

Data was analyzed in the same way as described in the previous section on the *LARGE1* haplotype MPRA.

Extended Figures and Tables

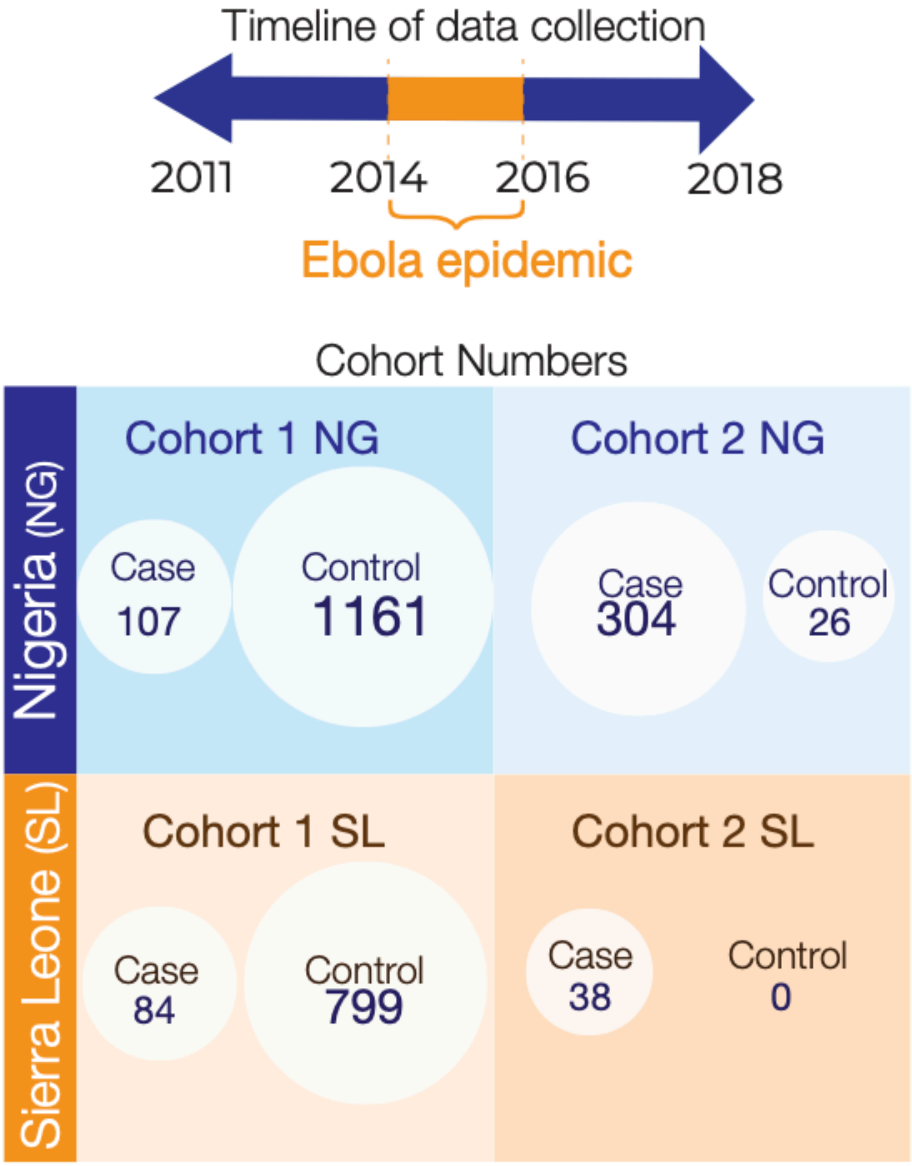

Extended Data Figure 1. Timeline of cohort recruitment in each country.
Breakdown of enrolled patients by country, cohort, and disease status.

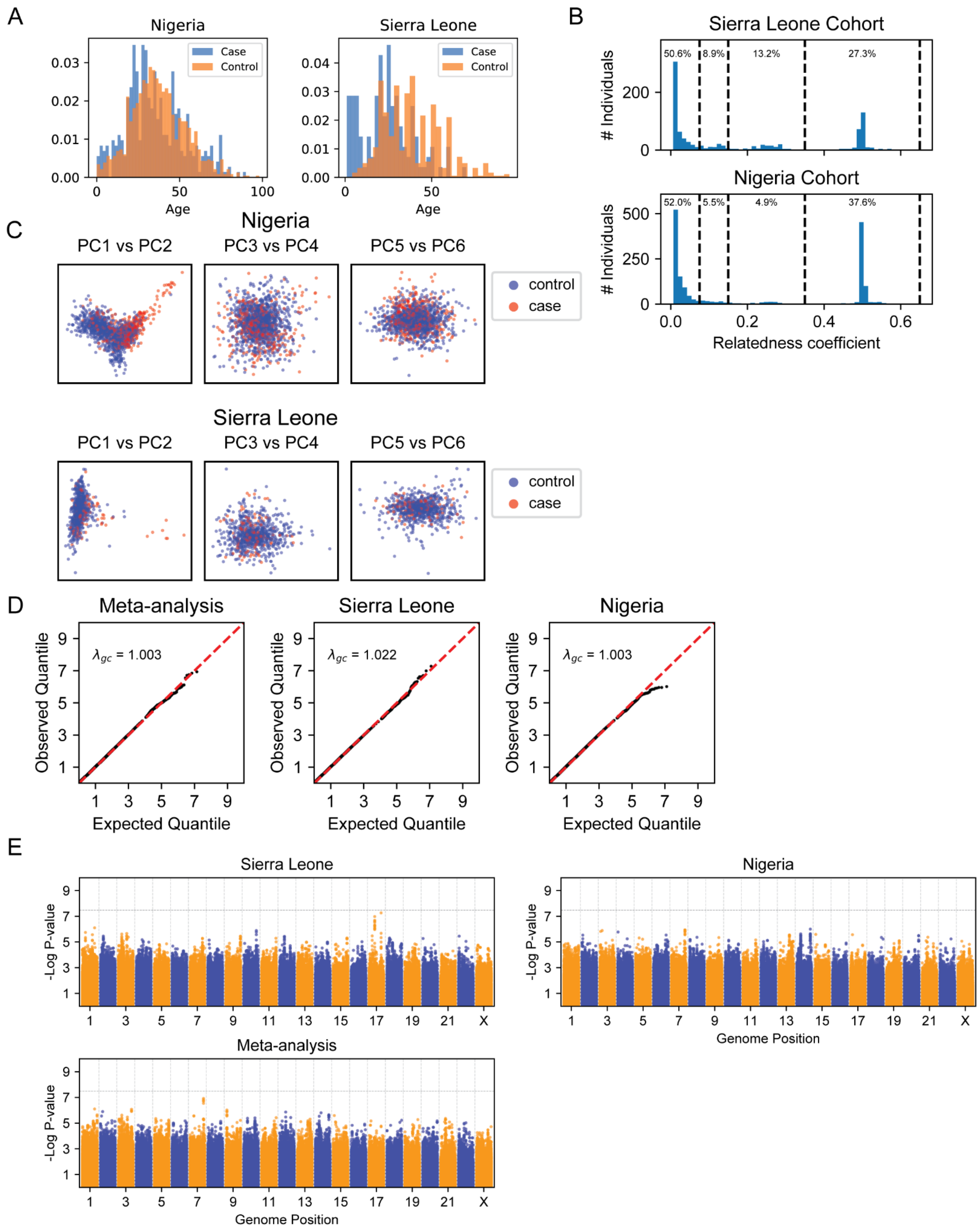
A
Nigeria
Sierra Leone
Case
Control
Age
B
Sierra Leone Cohort
Nigeria Cohort
Individuals
50.6%
8.9%
13.2%
27.3%
52.0%
5.5%
4.9%
37.6%
Relatedness coefficient
C
Nigeria
Sierra Leone
PC1 vs PC2
PC3 vs PC4
PC5 vs PC6
control
case
D
Meta-analysis
Sierra Leone
Nigeria
λ_{gc} = 1.003
λ_{gc} = 1.022
λ_{gc} = 1.003
Observed Quantile
Expected Quantile
E
Sierra Leone
Nigeria
Meta-analysis
-Log P-value
Genome Position

Extended Data Figure 2. Quality control analyses for the susceptibility GWAS. A) Histogram of ages in the Nigeria and Sierra Leone cohorts, separated by case/control status. B) Histogram of the maximum relatedness coefficient between each individual and all other individuals in the Nigerian (NG) and Sierra Leonean (SL) cohorts. C) Principal component analysis (PCA) of the NG and SL cohorts, colored by case-control status. PCs were computed on unrelated individuals and then all individuals were projected onto those components (Materials and Methods). D) Quantile-quantile plots of $-\log_{10}$ P-values from the susceptibility GWAS against expected quantiles. E) Manhattan plots showing the $-\log_{10}$ P-value for each genomic variant for the LF susceptibility associations. P-values in D and E are based on saddlepoint-approximated score tests (SAIGE), while meta-analysis P-values are derived from meta-analysis (METAL) of P-values generated from each cohort.

A

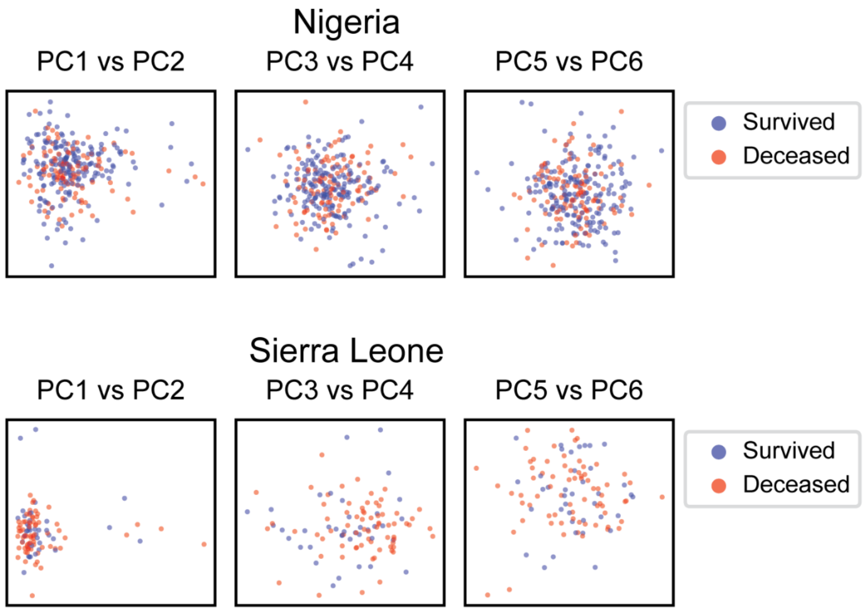

B

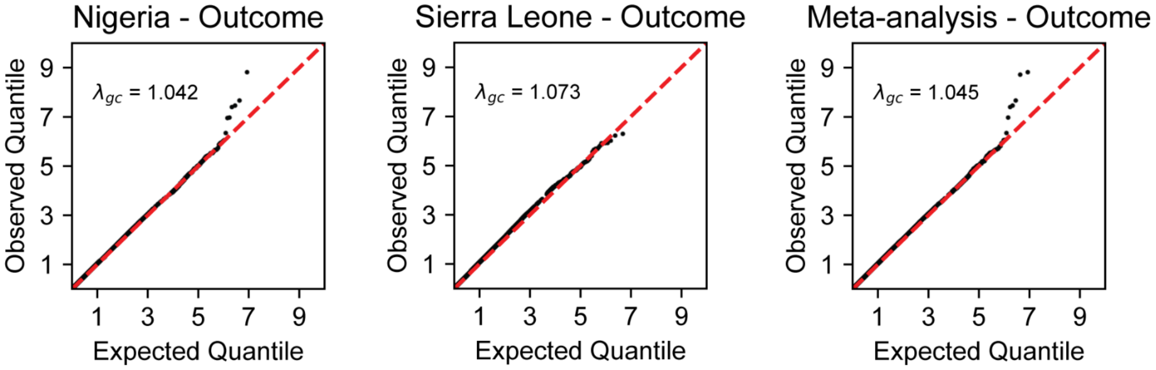

C

Variant Information			Without Age Covariate		With Age Covariate	
**Lead SNP**	**Nearest Gene**	**Cohort**	**OR**	**P-value**	**OR**	**P-value**
rs73404538	*LIF1*	NG	0.358	1.1×10^{-7}	0.359	2.2×10^{-8}
rs73404538	*LIF1*	SL	0.389	4.7×10^{-3}	0.389	8.6×10^{-3}
rs73404538	*LIF1*	Meta		1.9×10^{-9}		8.0×10^{-10}
rs9870087	*GRM7*	NG	15.4	1.5×10^{-9}	14.4	3.0×10^{-9}

Extended Data Figure 3. Quality control analyses for the GWAS of Lassa Fever clinical outcome. A) Principal component analysis (PCA) of the NG and SL cohorts, colored by clinical outcome. PCs were computed on unrelated individuals, and then all individuals were projected onto those components. B) Quantile-quantile plots of $-\log_{10}$ P-values from the outcome GWAS against expected quantiles. C) Comparison of the outcome GWAS lead variants with and without inclusion of age as a covariate. P-values in B and C are based on saddlepoint-approximated score tests (SAIGE), while meta-analysis P-values are derived from meta-analysis (METAL) of P-values generated from each cohort. Odds ratios are computed from Firth logistic regression.

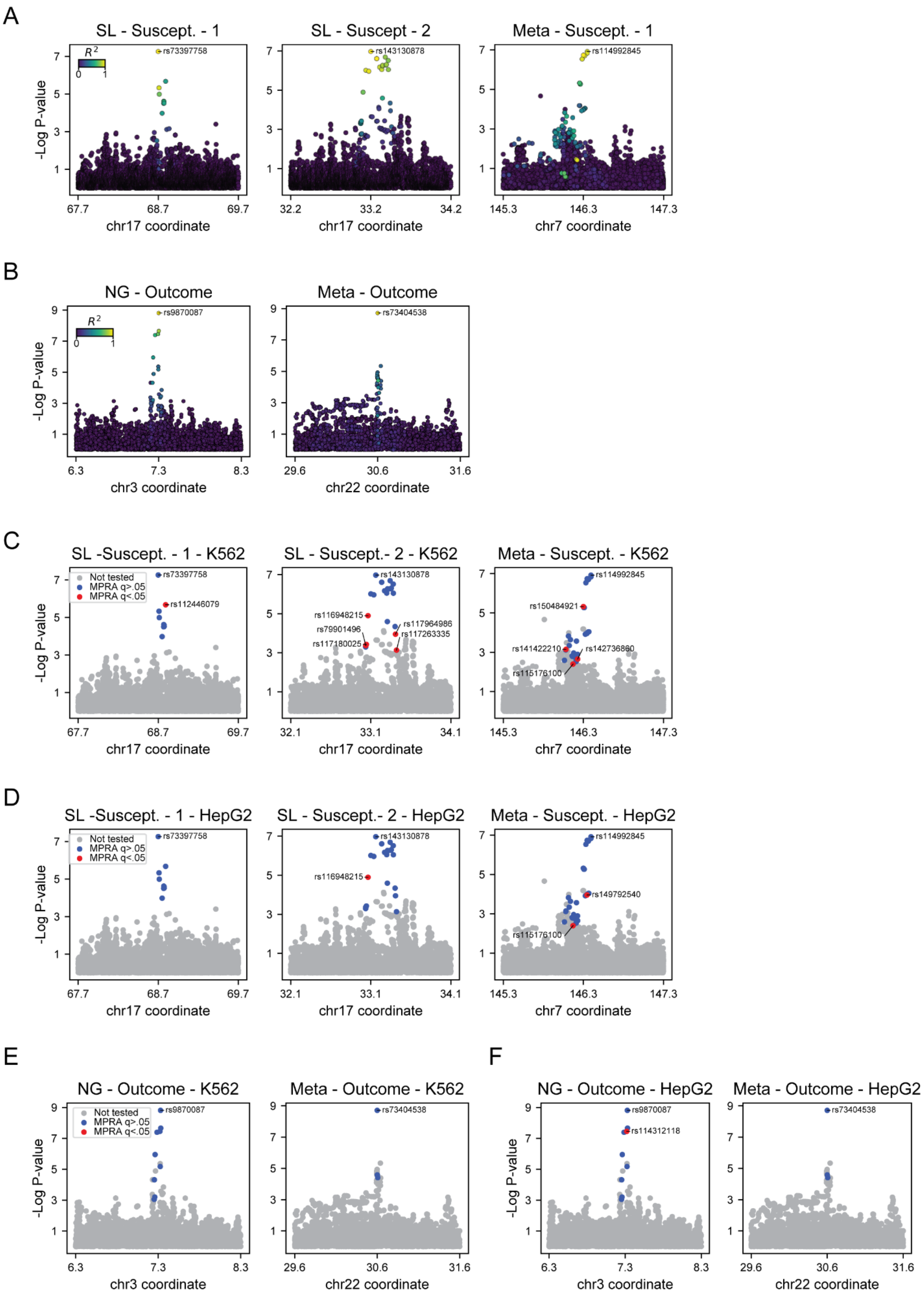
A
SL - Suscept. - 1
SL - Suscept - 2
Meta - Suscept. - 1
-Log P-value
R²
rs73397758
rs143130878
rs114992845
chr17 coordinate
chr7 coordinate
B
NG - Outcome
Meta - Outcome
rs9870087
rs73404538
chr3 coordinate
chr22 coordinate
C
SL -Suscept. - 1 - K562
SL - Suscept.- 2 - K562
Meta - Suscept. - K562
Not tested
MPRA q>.05
MPRA q<.05
rs112446079
rs116948215
rs79901496
rs117180025
rs117964986
rs117263335
rs150484921
rs141422210
rs142736860
rs115176100
D
SL -Suscept. - 1 - HepG2
SL - Suscept.- 2 - HepG2
Meta - Suscept. - HepG2
rs149792540
E
NG - Outcome - K562
Meta - Outcome - K562
F
NG - Outcome - HepG2
Meta - Outcome - HepG2
rs114312118

Extended Data Figure 4. MPRA analyses of the susceptibility and outcome GWAS peaks. A) Scatter plot of lead susceptibility GWAS loci described in the main text showing chromosomal position against $-\log_{10}$ association P-value. Variants are colored by the linkage disequilibrium (LD) coefficient of determination R^2 between each variant and the most significant "lead" variant in the locus. B) Same as A but for the lead variants in the fatal outcome GWAS. C-F) Same as A and B but colored by whether the variant showed statistically significant skew (q-value < 0.05) in the massively parallel reporter assay in the K562 cell line (C and E) or HepG2 cell line (D and F). P-values are based on saddlepoint-approximated score tests (SAIGE), while meta-analysis P-values are derived from meta-analysis (METAL) of P-values generated from each cohort.

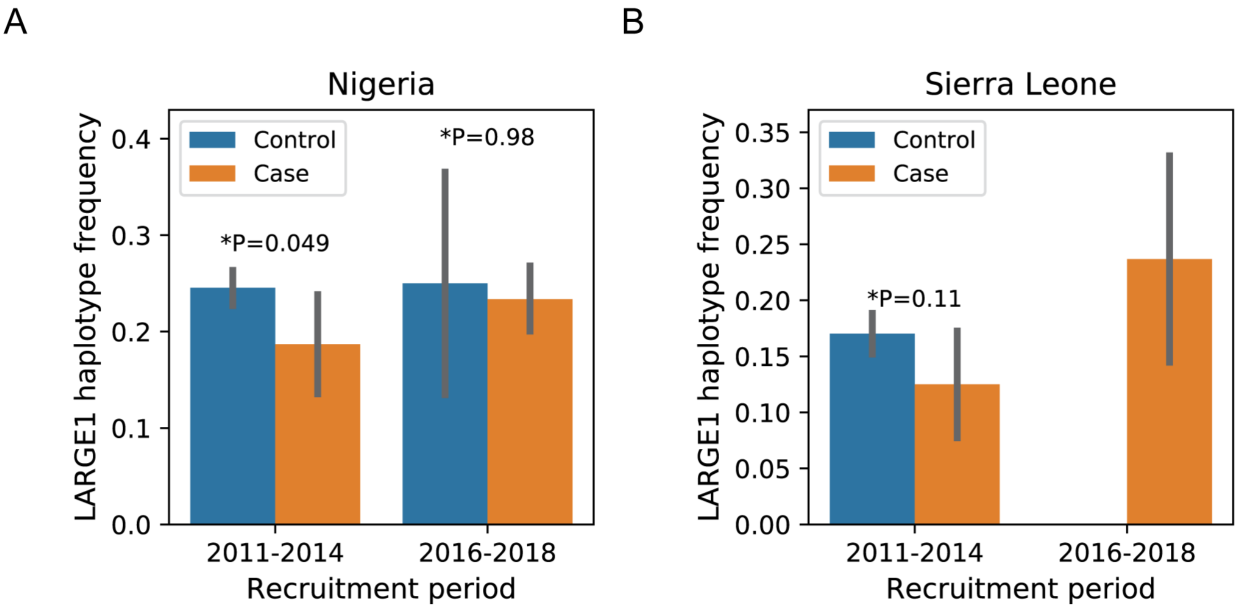

Extended Data Figure 5. *LARGE1* haplotype association by recruitment period. A,B) Frequencies of the long-range *LARGE1* haplotype by the period of recruitment as well as by case-control status for Nigeria (A) and Sierra Leone (B). P-values are from mixed logistic models association testing within the indicated recruitment period. Error bars represent 95% bootstrap confidence intervals for allele frequency. N for each cohort within each country is defined in Table S2.

Country	Phenotype	Total	Female (%)	Male (%)	Age Mean	Age SD	Deceased (%)	Survived (%)	Unknown (%)
Nigeria	Control	1187	497 (41.9)	690 (58.1)	37.2	16.1			
	Case	411	242 (58.9	169 (41.1)	35.2	17.9	107 (26.0)	196 (47.7)	108 (26.3)
Sierra Leone	Control	799	324 (40.6)	475 (59.4)	38.8	16.6			
	Case	122	50 (41.0)	72 (59.0)	24.1	14.9	70 (57.4)	38 (31.1)	14 (11.5)

Extended Data Table 1. Summary of GWAS collections for the Nigerian and Sierra Leonean cohorts. Includes breakdown of samples by sex, includes age mean and SD for each sample set, and breakdown of clinical outcome for cases.

Cohort		Count	Collection Period		Genotyping Array			Control Breakdown		Cases Diagnostics			
Country	Phenotype	Total	2011-2015 (%)	2016-2018 (%)	H3 (%)	Omni2.5M (%)	Omni5M (%)	Village Controls	Trio Controls	qPCR+ (%)	Antigen+ (%)	Seq+ (%)	Both+ (%)
Nigeria	Control	1187	1161 (97.8)	26 (2.2)	44 (3.7)	756 (63.7)	387 (32.6)	638 (53.7)	549 (46.3)				
	Case	411	107 (26.0)	304 (74.0)	317 (77.1)	91 (22.1)	3 (0.7)			119 (29.0)		114 (27.7)	178 (43.3)
Sierra Leone	Control	799	799 (100.0)	0 (0.0)	46 (5.8)	753 (94.2)	0 (0.0)	604 (75.6)	195 (24.4)				
	Case	122	84 (68.9)	38 (31.1)	38 (31.1)	84 (68.9)	0 (0.0)				80 (65.6)	11 (9.0)	31 (25.4)

Extended Data Table 2. **Detailed summary of GWAS collections.** Includes breakdown of samples by collection time period, genotyping array, split of controls into village and trio recruitments, and diagnostic categories of cases. For the case diagnostic category, Nigerian cases were positive by RT-qPCR (qPCR+) and/or sequencing (Seq+), whereas Sierra Leonean cases were positive by antigen ELISA (Antigen+) and/or sequencing. The last column Both+ specifies the number of cases who were positive by both sequencing and RT-qPCR or ELISA.

	Sierra Leone Cohort								Nigeria Cohort							
	Symptom Frequency By Age (%)						Statistics		Symptom Frequency By Age (%)						Statistics	
Symptom/Sign	0-9 (N=28)	10-19 (N=25)	20-29 (N=36)	30-39 (N=17)	40+ (N=16)	Total (N=122)	Z	P	0-9 (N=6)	10-20 (N=8)	20-30 (N=26)	30-40 (N=31)	40+ (N=38)	Total (N=109)	Z	P
Weakness	96.3	82.6	75	80	73.3	82.1	-2.01	0.045	16.7	25	53.8	45.2	42.1	43.1	0.78	0.435
Cough	96.3	78.3	75	53.3	66.7	76.8	-3.21	0.001	33.3	37.5	34.6	22.6	18.4	25.7	-1.77	0.077
Headache	63	87	75	73.3	93.3	76.8	1.87	0.062	16.7	12.5	76.9	61.3	50	55	0.6	0.546
Vomiting	81.5	73.9	56.2	66.7	46.7	66.1	-2.5	0.012	83.3	87.5	57.7	58.1	47.4	57.8	-2.4	0.016
Sore throat	55.6	69.6	62.5	60	60	61.6	-0.15	0.879	16.7	25	26.9	29	21.1	24.8	0.03	0.976
Abdominal pain	44.4	65.2	50	60	40	51.8	-0.04	0.967	50	37.5	46.2	58.1	47.4	49.5	0.29	0.769
Diarrhea	55.6	56.5	46.9	40	46.7	50	-1.47	0.141	16.7	12.5	30.8	16.1	21.1	21.1	-0.12	0.901
Fever	54.5	52.6	48	27.3	50	48.3	-1.18	0.237	33.3	87.5	42.3	58.1	47.4	51.4	-0.17	0.868
Bleeding	48.1	47.8	37.5	40	26.7	41.1	-1.35	0.177	16.7	25	30.8	29	15.8	23.9	-1.35	0.178
Swelling	40.7	34.8	37.5	33.3	6.7	33	-1.39	0.163								
Jaundice	3.7	8.7	3.1	26.7	6.7	8	1.26	0.207								
Injected conjun.									0	0	0	9.7	10.5	6.4	1.08	0.282
	0-9 (N=24)	10-19 (N=15)	20-29 (N=30)	30-39 (N=18)	40+ (N=21)	Total (N=108)	Z	P	0-9 (N=19)	10-19 (N=36)	20-29 (N=76)	30-39 (N=66)	40+ (N=98)	Total (N=295)	Z	P
Fatal outcome	45.8	73.3	66.7	72.2	71.4	64.8	1.36	0.174	26.3	19.4	36.8	36.4	42.9	35.3	1.61	0.107

Extended Data Table 3. **Overview of clinical symptoms.** Percentage of cases with a clinical sign or symptom at the time of admission, stratified by age. Below each age range is the number of individuals in that group with clinical data available. We report the large-sample approximation test statistic (Z) and P-value (P) for a Wilcoxon Ranksum test comparing the median age of subjects with and without each symptom. Conjunctival injection was recorded for the NG cohort but not the SL cohort, and lower extremity swelling or jaundice were recorded for the SL cohort but not the NG cohort. Bleeding includes any observed bleeding such as epistaxis, hematemesis, hematuria, melena, and hematochezia. Fever is defined as a temperature on admission of greater than 37.8 degrees celsius.

Variant Information			Susceptibility GWAS					Outcome GWAS				
Lead SNP	Chrom	Position (hg19)	Nigeria OR	Nigeria P-value	Sierra Leone OR	Sierra Leone P-value	Meta-analysis P-value	Nigeria OR	Nigeria P-value	Sierra Leone OR	Sierra Leone P-value	Meta-analysis P-value
rs114992845	7	146356694	9.19	2.7x10-6	4.77	0.010	1.2x10-7	1.14*	0.82*	8.24*	0.098*	0.18*
rs143130878	17	33192408	1.20	0.64	6.87	1.1x10-7	3.3x10-4	1.16	0.75	0.70*	0.56*	0.85*
rs73397758	17	68745251	0.84	0.58	9.16	5.5x10-8	4.8x10-3	0.56	0.14	22.6*	1.8x10-3*	0.25*
rs73404538	22	30619983	0.83	0.18	0.71	0.039	0.021	0.36	1.1x10-7	0.39	4.7x10-3	1.1x10-9
rs9870087	3	7330265	0.72	0.27	1.38*	0.46*	0.67*	15.4	1.5x10-9	0.64*	0.55*	1.1x10-6*

Extended Data Table 4. Comparison of lead variants between the outcome and susceptibility GWAS analyses. Displays odds ratios (OR) and P-values for lead variants in either the susceptibility GWAS (top) or outcome GWAS (bottom). P-values are based on saddlepoint-approximated score tests (SAIGE), while meta-analysis P-values are derived from meta-analysis (METAL) of P-values generated from each cohort. Odds ratios are computed from Firth logistic regression. *Variants with an asterisk were excluded from the corresponding analysis due to quality control filters but are included here for completeness.

Locus	Allele ID	Allele Frequency	Description
A	02:01@21	0.0019	One mismatch in exon 4; codon 245 position 3; GCG to GCA; Synonymous substitution (Ala to Ala)
B	35@1	0.0058	One mismatch in exon 5; codon 304 position1; GCT to ACT; Nonsynonymous substitution (Ala to Thr)
	15:10@23	0.0019	One mismatch in exon 3; codon 135 position 3; GCC to GCG; Synonymous substitution (Ala to Ala)
	35@24	0.0019	One mismatch in exon 3 codon 158, position 3; GCT to GCC; Synonymous substitution (Ala to Ala)
	53@25	0.0019	One mismatch in exon 3 codon 171, position 1; CAC to TAC; Nonsynonymous substitution (His to Tyr)
	42@26	0.0019	One mismatch in exon 3 codon 138, position 3; ACC to ACG; Synonymous substitution (Thr to Thr)
C	16:01@8	0.0019	One mismatch in exon 2; codon 62 position 3; CGG to CGA; Synonymous substitution (Arg to Arg)
	07@11	0.0019	One mismatch in exon 3 codon 100 position 3; GGT to GGC; Synonymous substitution (Gly to Gly)
	17@27	0.0019	One mismatch in exon 2; codon 105 position 3; CCG to CCC; Synonymous substitution (Pro to Pro)
DPA1	03:01@2*	0.05	One mismatch in exon 1 promoter region; codon -31 position 2; ATG to ACG; Nonsynonymous substitution (Met to Thr).
	03:01@3	0.0192	One mismatch in exon 4; codon 204 position 3; GTG to GTC; Synonymous substitution (Val to Val).
	02:07@4	0.0404	One mismatch in exon 4; codon 224 position 2; CGG to CAG; Nonsynonymous substitution (Arg to Gln).
	01@10	0.0019	One mismatch in exon 2; codon 20 position 3; GGA to GGG; Synonymous substitution (Gly to Gly).
	01:03@12	0.0019	One mismatch in exon 4; codon 204 position 3; GTG to GTC; Synonymous substitution (Val to Val).
	02:02@14	0.0077	One mismatch in exon 2; codon 38 position 3 AAA to AAG; Synonymous substitution (Lys to Lys).
	02@28	0.0019	Two mismatches in exon 2; codon 31 position 1 and position 2; CAG to ATG; Nonsynonymous substitution (Gln to Met)
DPB1	414:01@17	0.0019	One mismatch in exon 4; codon 205 position 1 ATG to GTG; Nonsynonymous substitution (Met to Val).
	333@20	0.0019	One mismatch in exon 2; codon 72 position 1 GTG to TTG; Nonsynonymous substitution (Val to Leu).
	01@29	0.0019	One mismatch in exon 2; codon 43 position 3; GGG to GGA; Synonymous substitution (Gly to Gly)
	26@30	0.0019	One mismatch in exon 4; codon 194 position 2; CAG to CGG; Nonsynonymous substitution (Gln to Arg)
DQA1	01:06@22	0.0019	One mismatch in exon 2; codon 44 position 1; GCT to ACT; Nonsynonymous substitution (Ala to Thr).
DQB1	04@5	0.0019	One mismatch in exon 3; codon 123 position 2; TAT to TGT; Nonsynonymous substitution (Tyr to Cys).
	05:02@6	0.0019	One mismatch in promoter region of exon 1; Position 544; A to G, non coding region.
	06@31	0.0019	One mismatch in exon 2; codon 9 position 2; TAC to TTC; Nonsynonymous substitution (Tyr to Phe)
	06@32	0.0038	One mismatch in exon 2; codon 48 position 3; CGC to CGG; Synonymous substitution (Arg to Arg)
	06@33	0.0019	One mismatch in exon 4; codon 224 position 2; CAG to CGG; Nonsynonymous substitution (Gln to Arg)

	06@34	0.0019	One mismatch in exon 2; codon 57 position 2; GTT to GAT; Nonsynonymous substitution (Val to Asp)
	06@35	0.0019	One mismatch in exon 3; codon 125 position 2; GGC to GCC; Nonsynonymous substitution (Gly to Ala)
	06@36	0.0019	Two mismatches in exon 2; codon 38 position 3; GCG to GCA; Synonymous substitution (Ala to Ala) and codon 47 position 3 TAT to TAC Synonymous substitution (Tyr to Tyr)
DRB1	13@37	0.0019	One mismatch in exon 2; codon 6 position 1; CGT to TGT; Nonsynonymous substitution (Arg to Cys)
	01@38	0.0019	One mismatch in exon 2; codon 74 position 3; GCC to GCG; Synonymous substitution (Ala to Ala)
DRB3	01:34@16	0.0038	One mismatch in exon 2; codon 85 position 2 GTT to GCT; Non synonymous substitution (Val to Ala).
	02:02@18	0.0038	One mismatch in exon 3; codon 113 position 3 AAC to AAA; Non synonymous substitution (Asn to Lys).
	02:02@19	0.0058	One mismatch in exon 2; codon 77 position 3 AAC to AAT; Synonymous substitution (Asn to Asn).
	01@39	0.0038	One mismatch in exon 2; codon 77 position 3; AAT to AAC; Nonsynonymous substitution (Ala to Gly)
	03@40	0.0038	Two mismatches in exon 2; codon 37 positions 1 and 2; TTC to AAC; Nonsynonymous substitution (Phe to Asn)
DRB4	01@13	0.0115	One mismatch in exon 2; Codon 32 position 3; TAC to TAT; (Synonymous substitution Tyr to Tyr).
	01@41	0.0019	One mismatch in exon 2; codon 76 position 2; GAC to GGC; Nonsynonymous substitution (Asp to Gly)
DRB5	02@7	0.0365	One mismatch in exon 4; codon 203 position 1; ATC to GTC; Nonsynonymous substitution (Ile to Val).
	02@9	0.0019	One mismatch in exon 2; codon 67 position 1; TTC to ATC; Nonsynonymous substitution (Phe to Ile).
	02@15	0.0077	2 mismatches: 1) exon 3; codon 138 position 1; GAG to AAG; Nonsynonymous substitution (Glu to lys) 2) exon 4; codon 203 position 1; ATC to GTC; Nonsynonymous substitution (Ile to Val).

Extended Data Table 5. Uncovered HLA alleles. Novel HLA alleles identified in sequence-based HLA typing of 297 Sierra Leoneans.

Supplementary Data Table legends

Tables 1-2 provide the P-values and estimated meta-analysis Z-scores for the susceptibility and outcome GWASes respectively. Table 3 and 4 provide the MPRA results data for the K562 and HepG2 cell lines for the lead GWAS association peaks, respectively. Table 5 provides the analogous MPRA data for the *LARGE1* long-range haplotype. For tables 3-5, statistical significance was assessed using a negative binomial generalized linear model, and standard errors were derived from Wald tests.

72. Olschläger, S. *et al.* Improved detection of Lassa virus by reverse transcription-PCR targeting the 5’ region of S RNA. *J. Clin. Microbiol.* **48**, 2009–2013 (2010).
73. Nikisins, S. *et al.* International external quality assessment study for molecular detection of Lassa virus. *PLoS Negl. Trop. Dis.* **9**, e0003793 (2015).
74. Boisen, M. L. *et al.* Field evaluation of a Pan-Lassa rapid diagnostic test during the 2018 Nigerian Lassa fever outbreak. *Sci. Rep.* **10**, 8724 (2020).
75. Chen, H. *et al.* Control for Population Structure and Relatedness for Binary Traits in Genetic Association Studies via Logistic Mixed Models. *Am. J. Hum. Genet.* **98**, 653–666 (2016).
76. Consortium, T. H. R. & the Haplotype Reference Consortium. A reference panel of 64,976 haplotypes for genotype imputation. *Nature Genetics* vol. 48 1279–1283 Preprint at https://doi.org/10.1038/ng.3643 (2016).
77. Loh, P.-R. *et al.* Reference-based phasing using the Haplotype Reference Consortium panel. *Nat. Genet.* **48**, 1443–1448 (2016).
78. Carrasco Pro, S. *et al.* Widespread perturbation of ETS factor binding sites in cancer. *Nat. Commun.* **14**, 913 (2023).
