# Supplementary material for: Genome-wide association study identifies human genetic variants associated with fatal outcome from Lassa fever
Source: Nat Microbiol. 2024 Feb 7;9(3):751–62. doi: 10.1038/s41564-023-01589-3 (PMC10914620; doi:10.1038/s41564-023-01589-3)
Supplement: Supplementary file 2 — Reporting Summary [file 41564_2023_1589_MOESM2_ESM.pdf]

nature portfolio

Corresponding author(s): Dylan Kotliar, Pardis C. Sabeti, Christian Happi

Last updated by author(s): August 29, 2023

Reporting Summary

Nature Portfolio wishes to improve the reproducibility of the work that we publish. This form provides structure for consistency and transparency in reporting. For further information on Nature Portfolio policies, see our Editorial Policies and the Editorial Policy Checklist.

Statistics

For all statistical analyses, confirm that the following items are present in the figure legend, table legend, main text, or Methods section.

n/a	Confirmed	
☐	☒	The exact sample size (*n*) for each experimental group/condition, given as a discrete number and unit of measurement
☐	☒	A statement on whether measurements were taken from distinct samples or whether the same sample was measured repeatedly
☐	☒	The statistical test(s) used AND whether they are one- or two-sided<br>*Only common tests should be described solely by name; describe more complex techniques in the Methods section.*
☐	☒	A description of all covariates tested
☐	☒	A description of any assumptions or corrections, such as tests of normality and adjustment for multiple comparisons
☐	☒	A full description of the statistical parameters including central tendency (e.g. means) or other basic estimates (e.g. regression coefficient) AND variation (e.g. standard deviation) or associated estimates of uncertainty (e.g. confidence intervals)
☐	☒	For null hypothesis testing, the test statistic (e.g. *F*, *t*, *r*) with confidence intervals, effect sizes, degrees of freedom and *P* value noted<br>*Give P values as exact values whenever suitable.*
☒	☐	For Bayesian analysis, information on the choice of priors and Markov chain Monte Carlo settings
☒	☐	For hierarchical and complex designs, identification of the appropriate level for tests and full reporting of outcomes
☐	☒	Estimates of effect sizes (e.g. Cohen's *d*, Pearson's *r*), indicating how they were calculated

Our web collection on statistics for biologists contains articles on many of the points above.

Software and code

Policy information about availability of computer code

Data collection Analysis Software	Illumina GenomeStudio version 2.0 was used to call genotypes from the raw array images. Illumina Assign 2.0 TruSight HLA was used to call HLA alleles from long read sequencing data.
Data analysis	The Sanger Imputation Service with Eagle2 phasing (as implemented here: https://imputation.sanger.ac.uk/?about=1) was used to impute non-genotyped variants from the genotyping data. SAIGE version 1.2.0 was used to perform association testing. The HIBAG version 1.22 software was used for imputing HLA alleles from SNP genotyping data. We used version 1.2.0 of SAIGE to conduct all genetic association tests. We used the GMMAT R package to identify variants with significant differences in allele frequency between groups (available at https://github.com/hanchenphd/GMMAT). Custom analysis scripts used in this manuscript are available on Github at https://github.com/dylkot/lassa_fever_gwas. Data from the MPRA was analyzed using MPRAmatch, MPRAcount, and MPRAmodel, available on https://github.com/tewhey-lab/MPRA_oligo_barcode_pipeline and https://github.com/tewhey-lab/MPRAmodel.

For manuscripts utilizing custom algorithms or software that are central to the research but not yet described in published literature, software must be made available to editors and reviewers. We strongly encourage code deposition in a community repository (e.g. GitHub). See the Nature Portfolio guidelines for submitting code & software for further information.

Data

Policy information about availability of data

All manuscripts must include a data availability statement. This statement should provide the following information, where applicable:

- Accession codes, unique identifiers, or web links for publicly available datasets
- A description of any restrictions on data availability
- For clinical datasets or third party data, please ensure that the statement adheres to our policy

Raw de-identified genetic data from this study has been uploaded to the European Genome-Phenome Archive under dataset ID EGAD00010002510 and EGAD00010002509, and can be accessed t https://ega-archive.org/register/. Summary statistics for the genetic analyses performed in this study are included as extended data tables 1-6 and summary statistics for the massively parallel reporter assay are included as extended data tables 7-9. Data from the 1000 Genomes Project is available at https://www.internationalgenome.org/data/. Genome assembly hg19 is available at https://www.ncbi.nlm.nih.gov/datasets/genome/GCF_000001405.13/.

Research involving human participants, their data, or biological material

Policy information about studies with human participants or human data. See also policy information about sex, gender (identity/presentation), and sexual orientation and race, ethnicity and racism.

Reporting on sex and gender

Self-reported gender was collected during study collection. In addition, biological sex was determined through analysis of the genetic data. Biological sex was used as a covariate in the GWAS.

Reporting on race, ethnicity, or other socially relevant groupings

We report the country of origin of all participants as being from Nigeria or Sierra Leone. In addition, for participants recruited jointly with the 1000 Genomes Project, we report the tribal affiliation of the participant as Yoruba, Esan, or Mende.

Population characteristics

- Age (years)
- Biological Sex
- SNP genotyping
- Lassa Virus IgG serostatus
- Genetic ancestry as reflected in principal component analyses
- Directly typed or imputed HLA alleles for class I and class II genes
- Lassa Fever case or population control status
- Clinical outcome from Lassa Fever (survival or fatal outcome)
- Clinical symptoms from Lassa Fever

Recruitment

Lassa Fever case definition and recruitment
Irrua Specialist Teaching Hospital (ISTH), Nigeria

We recruited LF cases at ISTH between 2011-2014 and 2016-2018 with a gap from 2014-2016 due to the Ebola outbreak in West Africa that temporarily halted research operations. We performed molecular diagnostic testing for all suspected cases who met clinical diagnostic criteria for LF including fever >38 °C for less than 3 weeks, absence of signs of local inflammation, absence of clinical response to anti-malarials, and additional major and minor signs. Suspected cases who were positive by molecular diagnostic testing were recruited to the study following informed consent.

From 2011 to 2014, ISTH study staff performed RT-PCR targeting the GPC gene62 as the primary diagnostic and positive cases were recruited into the study. However, due to concerns about false positives of this initial assay, a confirmatory RT-qPCR assay was performed at the Broad institute in Boston using primers against the LASV S segment (forward: CCCAAGCYCTHCCYACAAT, reverse: AACCCTTATGAGAAYATACTBTAYAA) and a subset of patients underwent next-generation viral sequencing12. We only included data from recruited cases who were positive by this latter RT-qPCR or who had positive LASV sequencing with greater than 1 viral reads per kilobase (RPKM) in the GWAS analysis.

Between 2016-2018, ISTH patients who met clinical diagnostic criteria for LF were tested at ISTH with 2 RT-qPCR assays, one targeting the GPC gene (RealStar LASV RT-PCR Kit 1.0 CE, Altona Diagnostics, Hamburg, Germany) and a second targeting the LASV L segment63,64. Suspected cases from this period who were positive by either RT-qPCR assay were recruited to the study following informed consent. A subset of these cases also underwent viral sequencing26. We only included data from suspected LF cases who were positive by both of the RT-qPCR assays, or by viral genomic sequencing (with > 1 RPKM from the viral genome) in the GWAS.

Kenema Government Hospital (KGH), Sierra Leone

LF cases were recruited at KGH between 2011 and 2018 with a gap from 2015-2016 due to the Ebola outbreak in West Africa. Suspected cases included individuals who met clinical diagnostic criteria for LF24 and were positive by either ELISA for a LASV antigen or IgM antibody against LASV28,65. We additionally performed viral sequencing from a subset of enrolled cases12. We only included data from suspected cases who were either antigen-ELISA positive or viral sequencing positive with RPKM > 1 in the GWAS.

Population control recruitment

Study staff at ISTH and KGH recruited population controls through outreach efforts to villages with a recent history of LF cases. Village controls (Table S1) were healthy individuals who were recruited from the same household and/or village as cases, prioritizing unrelated individuals where possible. Trio controls (Table S1) were healthy families of mother, father, and

child from the Esan population in NG and the Mende population in SL who were recruited jointly with Phase 3 of the 1000 Genomes Project (1KGP).
Given that very few Lassa Fever cases are documented annually and mostly occur in rural areas far from diagnostic centers, we are likely identifying only the fraction of cases in which extreme disease presentations motivated patients to seek medical attention; results are thus more likely to reflect aspects of severe cases than subclinical ones.

Ethics oversight	This work was evaluated and approved under the purview of the following institutional review boards / local ethics committees: Nigerian National Health Research Ethics Committee and Irrua Specialist Teaching Hospital (ISTH/HREC/20170915/22), Sierra Leone Ethics and Scientific Review Committee (070716), Tulane University Human Research Protections Office (10-191330), and Harvard University Area Committee on the Use of Human Subjects (19-0023). Enrollment procedures and sampling efforts were executed at ISTH, KGH, and their surrounding communities with participant consent or through a waiver of consent granted by the appropriate institutional review board / local ethics committee. A subset of samples shared with the study collaboration include those stored as clinical excess or approved for secondary use.

Note that full information on the approval of the study protocol must also be provided in the manuscript.

Field-specific reporting

Please select the one below that is the best fit for your research. If you are not sure, read the appropriate sections before making your selection.

☒ Life sciences ☐ Behavioural & social sciences ☐ Ecological, evolutionary & environmental sciences

For a reference copy of the document with all sections, see nature.com/documents/nr-reporting-summary-flat.pdf

Life sciences study design

All studies must disclose on these points even when the disclosure is negative.

Sample size	Power calculations suggested >90% power to obtain genome-wide significance for an effect variant with minor allele frequency greater than 0.15 and genomic risk ratio greater than 2.00. Our case-control design is intended to detect causal resistance alleles that are under positive selection, thus power was determined for common variants with a larger effect-size than most variants affecting complex traits.
Data exclusions	Data was excluded for recruited individuals who did not meet our case definitions. This includes suspected Lassa Fever cases based on symptoms who did not meet the molecular diagnostic criteria for definition as a Lassa Fever case. In addition, data was excluded when the genotype-determined sex did not match the self-reported sex of the study participant as these likely reflected sample swap errors.
Replication	We recruited Lassa Fever cases and controls from two study sites, in Nigeria and Sierra Leone, to serve as replication cohorts. rs181032423 had a genome-wide significant association with susceptibility in the Nigerian cohort (P = 2.2x10-8) and was also nominally associated with susceptibility in the Sierra Leone cohort (P = 0.026). Other findings were significant in only one or the other cohort which could be due to viral genetic diversity between the two countries.
Randomization	This was a case-control study design and did not involve randomization.
Blinding	As this was a case-control study design, investigators were not blinded to group allocation.

Reporting for specific materials, systems and methods

We require information from authors about some types of materials, experimental systems and methods used in many studies. Here, indicate whether each material, system or method listed is relevant to your study. If you are not sure if a list item applies to your research, read the appropriate section before selecting a response.

Materials & experimental systems

n/a	Involved in the study	
☒	☐	Antibodies
☒	☐	Eukaryotic cell lines
☒	☐	Palaeontology and archaeology
☒	☐	Animals and other organisms
☒	☐	Clinical data
☐	☒	Dual use research of concern
☒	☐	Plants

Methods

n/a	Involved in the study	
☒	☐	ChIP-seq
☒	☐	Flow cytometry
☒	☐	MRI-based neuroimaging

Dual use research of concern

Policy information about dual use research of concern

Hazards

Could the accidental, deliberate or reckless misuse of agents or technologies generated in the work, or the application of information presented in the manuscript, pose a threat to:

No	Yes	
☐	☒	Public health
☒	☐	National security
☒	☐	Crops and/or livestock
☒	☐	Ecosystems
☒	☐	Any other significant area

Hazards: Blood samples were collected from patients with Lassa Fever, a transmissible and deadly virus.

For examples of agents subject to oversight, see the United States Government Policy for Institutional Oversight of Life Sciences Dual Use Research of Concern.

Experiments of concern

Does the work involve any of these experiments of concern:

No	Yes	
☒	☐	Demonstrate how to render a vaccine ineffective
☒	☐	Confer resistance to therapeutically useful antibiotics or antiviral agents
☒	☐	Enhance the virulence of a pathogen or render a nonpathogen virulent
☒	☐	Increase transmissibility of a pathogen
☒	☐	Alter the host range of a pathogen
☒	☐	Enable evasion of diagnostic/detection modalities
☒	☐	Enable the weaponization of a biological agent or toxin
☒	☐	Any other potentially harmful combination of experiments and agents

Precautions and benefits

Biosecurity precautions	All samples were stored in locked freezers and laboratories for the full extent of their existence and were chemically inactivated as soon as it was reasonable to do so.
Biosecurity oversight	All work was conducted in accordance with the overseeing biosafety committees of the institutes where work took place.
Benefits	This work will lead to a better understanding of the host genetic factors that impact susceptibility to Lassa Fever, which may eventually aid in the development of vaccines and other therapeutics.
Communication benefits	We don't see any major risks in communicating the findings in this study.
